# Physiological Effect of Thallium in the Facultative Hyperaccumulator *Silene latifolia*

**DOI:** 10.1111/ppl.70469

**Published:** 2025-08-27

**Authors:** Gaia Regini, Isabella Bettarini, Ilaria Colzi, Emilio Corti, Alessio Papini, Marco Dainelli, Giorgia Guardigli, Antony van der Ent, Nadia Bazihizina, Cristina Gonnelli

**Affiliations:** ^1^ Department of Biology Università degli Studi di Firenze Florence Italy; ^2^ Laboratory of Genetics Wageningen University and Research Wageningen the Netherlands

**Keywords:** chlorophyll fluorescence, hyperaccumulation, leaf gas exchange, metallophytes, stomata

## Abstract

The metallicolous populations of the facultative Tl hyperaccumulator *Silene latifolia* are extraordinarily tolerant and capable of accumulating up to 80,000 μg Tl g^−1^ in nature. A growth stimulatory effect of Tl was observed, and this study set out to determine possible mechanisms. Plants from non‐metallicolous and metallicolous populations were subjected to hydroponics dosing experiments at 2.5 and 10 μM Tl. Metal impact on stomatal and non‐stomatal photosynthetic constraints, light energy conversion processes and plant anatomy/ultrastructure was assessed over time. Photosynthetic rates improved in 10 μM Tl‐treated metallicolous plants by 20% compared to controls, partly due to increased stomatal conductance. The latter was mainly driven by Tl‐induced anatomical changes, such as increased central cylinder area and stomatal density, likely to enhance water uptake/translocation and, consequently, leaf metal accumulation. The apparently Tl‐favoured CO_2_ trafficking resulted in ameliorated maximal photosynthetic capacity. The first signs of photosynthetic declines appeared only at very high Tl leaf concentrations (15,000 μg Tl g^−1^), with limitations involving stomatal and biochemical factors; whereas the photochemical reactions remained functional. The observed Tl‐induced stimulatory response in growth and net photosynthetic rate in metallicolous plants shows that Tl improves physiological performance in *Silene latifolia,* mainly through improved stomatal conductance.

## 1 Introduction

Thallium (Tl) pollution has recently become an environmental concern worldwide (Xiao et al. 2004; EPA 2015). Most soils have low concentrations (< 1.7 mg Tl kg^−1^), but areas with geogenic Tl anomalies also occur with > 10 mg Tl kg^−1^ (Kazantzis 2000; Zhang et al. 2000). The increasing release of this metal in the environment is primarily driven by mining activities in which Tl forms an impurity (Cobelo‐García et al. 2015). In nature, Tl occurs in two oxidation states, with the monovalent form (Tl^+^) dominant over the trivalent form (Tl^3+^) (Karbowska 2016; Migaszewski and Gałuszka 2021). The toxicity of Tl^+^ is linked to its chemical characteristics, as it interferes with K‐dependent biochemical processes given their similar ionic radii and it forms toxic complexes with sulfhydryl groups of different biomolecules (Babula et al. 2008; Chang and Chiang 2024). In plants, Tl is found at low tissue concentrations, typically < 0.1 μg Tl g^−1^ dry weight (Markert et al. 2013), with a remarkably low toxicity threshold of around 20 μg Tl g^−1^ (Kabata‐Pendias and Pendias 2001). Thallium can therefore be considered one of the most toxic elements, surpassing even arsenic or mercury in toxicity (Kazantzis 2000; Lennartson 2015; Kemnic and Coleman 2025).

As Tl pollution continues to spread in the environment, research on Tl‐induced effects on living organisms has progressively grown in the last decades, leading to the identification of some of its toxicity mechanisms on plants. These include the induction of oxidative stress, DNA damage, impairment of K homeostasis and alterations in nutrient accumulation (Radić et al. 2009; Espinosa et al. 2023; Chang et al. 2024). Several studies have also highlighted the impact of Tl on photosynthetic activity, with excess Tl disrupting thylakoid structure, reducing chlorophyll fluorescence and concentration and lowering net photosynthetic rate and stomatal opening (Carlson et al. 1975; Naumann et al. 2007; Domínguez et al. 2011; Mazur et al. 2016). Thallium can downregulate the expression of genes linked to the Light‐harvesting complex II (LHCII), thereby affecting the photosynthetic process during solar energy harvesting (Chang et al. 2024). The effect of Tl excess on the photosynthetic machinery has been mainly studied in model plants, which typically respond to the excess of any metal by limiting its translocation to the shoots (Tang et al. 2023). No information is available on the effect of Tl on the photosynthetic activity in the extraordinarily rare plant species capable of hyperaccumulation.

Hyperaccumulators are plant species that accumulate metals or metalloids in their shoots at extremely high concentrations (van der Ent et al. 2013), with this trait generally considered a mechanism for protection against herbivores and pathogens (Poschenrieder et al. 2006). Thallium hyperaccumulation is recognised at foliar concentrations exceeding 100 μg Tl g^−1^ (van der Ent et al. 2013; Reeves et al. 2018), and Tl hyperaccumulators are among the rarest of all known hyperaccumulator plants (van der Ent et al. 2013). Only a few European plant species have been identified as such, originating from Zn‐Pb mines in Southern France and Northern Italy (Escarré et al. 2011; Fellet et al. 2012) and from the Tl‐As outcrop at Allchar in North Macedonia (Jakovljević et al. 2024). The search for new Tl hyperaccumulators is, however, very promising, as more species are likely to be discovered in scarcely explored Tl‐enriched areas, such as the Lanmuchang site in China (Zhang et al. 2000). Scientific research on Tl hyperaccumulator plants has gained significant momentum in recent years. This interest is driven not only by the biologically striking discovery of the highest metal concentration ever recorded in plants (around 80,000 μg Tl g^−1^, Jakovljević et al. 2025), but also by the potential for eco‐friendly and economically viable Tl phytoextraction from contaminated soils (Robinson and Anderson 2021). The emerging research on these plants has provided insights into the mechanisms of Tl hyperaccumulation, including vacuolar compartmentalisation and specific leaf tissue localization (Corzo Remigio, Pošćić, et al. 2022; Corzo Remigio, Nkrumah, et al. 2022; Salinitro et al. 2024), detoxification via extracellular crystalline deposits of TlCl around the vascular bundles (Corzo Remigio et al. 2025; Salinitro et al. 2024), enhanced ionome stability under Tl treatment and the possible involvement of sulphur‐containing compounds in metal detoxification (Regini et al. 2024).

Given the extraordinary level of Tl accumulation in hyperaccumulator plant leaves, one key question that has yet to be addressed is how these extreme Tl concentrations affect photosynthetic parameters in these species. When considering the impact of other elements (e.g., Cd, Zn and Ni) in long‐discovered hyperaccumulators, evidence suggests that the excess of metals can either negatively affect stomatal and non‐stomatal photosynthetic processes, as in the case of Cd and Zn, or improve them, as in the case of Ni, depending on the species investigated (Zhou and Qiu 2005; Küpper et al. 2007; Meyer et al. 2010; Ying et al. 2010; Moustakas et al. 2019; Szopiński et al. 2019, 2020; Scartazza et al. 2022; Colzi et al. 2023). Thus, given that studies on non‐accumulators have shown the inhibitory effect of Tl on photosynthesis, it remains to be determined whether hyperaccumulators experience similar limitations, albeit at higher tissue concentrations, or if they exhibit different, species‐specific physiological adaptations. Currently, the only available information is on the chlorophyll fluorescence parameter F_V_/F_M_, reported to suffer negligible effects under Tl treatment in the Tl hyperaccumulator *Silene latifolia* (Regini et al. 2024). *Silene latifolia* Poir. subsp. *alba* (Mill.), widely distributed in Europe, Western Asia and Northern Africa, is a facultative hyperaccumulator (Pollard et al. 2014) that expresses Tl hyperaccumulation only in metallicolous populations and not in non‐metallicolous populations (Corzo Remigio, Nkrumah, et al. 2022; Regini et al. 2024). Field‐collected samples at the Allchar site in Northern Macedonia have recently recorded *S. latifolia* as the species with the highest Tl concentration ever recorded in a living organism, with up to 79,200 μg Tl g^−1^ in leaves (Jakovljević et al. 2024). Therefore, this species is an excellent model to study the physiology of Tl hyperaccumulation and the influence of Tl on the photosynthetic process, while this species also holds promise for Tl phytoextraction from polluted soils (Corzo Remigio et al. 2020; Corzo Remigio, Nkrumah, et al. 2022).

In this study, the impact of Tl on stomatal and non‐stomatal photosynthetic constraints, light energy conversion processes and plant anatomy and ultrastructure was compared between a metallicolous and a non‐metallicolous population of *S. latifolia* . The aim was to test the hypothesis that Tl not only differentially affected the analysed photosynthetic traits in two contrasting groups of plants but also improved them in the hyperaccumulating population. This is because the Tl levels accumulated by the metallicolous *S. latifolia* accession are so high that they fall within the concentration range of K typically occurring at 20,000–50,000 μg g^−1^ in most plants (Marschner 1995). This, therefore, raises the possibility that Tl might have a beneficial physiological role in the hyperaccumulating metallicolous accession of *S. latifolia*.

## 2 Materials and Methods

### 2.1 Plant Growth in Hydroponics and Tl Treatments

Seeds of *Silene latifolia* Poir. subsp. *alba* (Mill.) were collected from natural populations in July 2023 in France. The non‐metallicolous accession was collected from Barraux (45°26′0″ N, 5°59′0″ E) from non‐contaminated soil and the metallicolous accession was collected from Saint‐Laurent‐le‐Minier (43°55′55″ N, 3°39′19″ E) from Zn‐Pb‐Cd‐Tl contaminated soil (for further information see Regini et al. 2024). The metallicolous population evolved on a Tl‐enriched substrate (Escarré et al. 2011) and according to the idea that metal excess in the environment causes strong local selective pressures (Antonovics et al. 1971), appeared to be Tl hyper‐tolerant and hyperaccumulating in contrast to the non‐metallicolous one (Regini et al. 2024). The seeds were sown in peat soil, and after 15 days, the seedlings were transferred to hydroponic cultures. Each plant was placed in a 1‐L polyethylene pot with modified half‐strength Hoagland's solution composed as follows: 3 mM KNO_3_, 2 mM Ca(NO_3_)_2_, 1 mM NH_4_H_2_PO_4_, 0.50 mM MgSO_4_, 20 μM Fe(Na)‐EDTA, 1 μM KCl, 25 μM H_3_BO_3_, 2 μM MnSO_4_, 2 μM ZnSO_4_, 0.1 μM CuSO_4_ and 0.1 μM (NH_4_)_6_Mo_7_O_24_ (Hoagland and Arnon 1950) in milliQ‐water (Millipore) buffered with 2 mM 2‐morpholinoethanesulphonic acid, adjusted to pH 5.5 with KOH. Experiments were conducted in a growth chamber with a 16 h (day) photoperiod, day/night temperature of 24/16°C, an irradiance of 300 μmol m^−2^ s^−1^ and 60%–65% relative humidity. After a pre‐culture period of 7 d, plants of equal size were selected for the treatment (mean root length and leaf area: 12.1 ± 1.6 cm and 17.7 ± 2.8 cm^2^ for the non‐metallicolous population and 10.4 ± 1.5 cm and 12.1 ± 2.0 cm^2^ for the metallicolous population, respectively. Values are means of 36 replicates ± standard deviation) and exposed to 2.5 and 10 μM Tl supplied as TlNO_3_ (Sigma‐Aldrich) in a background solution with the same composition as the pre‐culture solution. Treatment concentrations were chosen based on previous research (Regini et al. 2024). Plants were sampled at the beginning of the treatment and after 4, 8 and 12 days of exposure. At each time point, 12 plants per treatment from each population were collected for root length and leaf area measurements to monitor growth increment at every interval (calculated by subtracting length or area values at the beginning of the treatment from values at the first, the second or the third interval, as in Colzi et al. 2023). To measure leaf area, pictures from above were taken using a digital camera (Canon PowerShot SX100 IS), accurately placed at a distance of 40 cm from the bottom of the pots. The Fiji software ImageJ (Schindelin et al. 2012) was used for the image analysis, and the rosette area was expressed in cm^2^. After the biometric measurements, roots were gently washed with 1 mM EDTA at 4°C for 15 min to desorb metals adhering to the cell wall, as in Körner et al. (1987). Roots and shoots were dried to constant weight at 40°C for 72 h prior to elemental analysis.

### 2.2 Thallium Concentrations

Root and shoot Tl concentrations were determined using monochromatic X‐ray fluorescence (XRF) analysis (E‐max Ultra, Z‐Spec Inc.) as described in Regini et al. (2024). Each sample was ground using a mortar and pestle to obtain a fine powder. Approximately 100 mg of powder was placed in an XRF sample cup and covered with a polypropylene thin film (12 μm) and analysed for 60 s three times while rotating the sample cup in different positions each time (the values of the three replicate measurements were averaged). The following certified reference materials were used to calibrate the instrument, were used: NIST 1568b rice flour, NIST 1567b wheat flour, NMIJ 7502a white rice flour, NIST 1570a spinach leaves and NIST 1573a tomato leaves.

### 2.3 Determination of Leaf Relative Water Contents

At the conclusion of the hydroponic experiment, one mature leaf from each plant was excised and immediately weighed, placed in distilled water for 4 h to reach full turgidity and then weighed again. Samples were then dried in an oven at 40°C for 72 h, and the dry weight was recorded. Water Content and Relative Water Content were calculated as follows: WC (%) = [(FW−DW)/FW] × 100 and RWC (%) = [(FW−DW)/(TW−DW)] × 100 (Pieczynski et al. 2012), where FW, DW and TW were the leaf fresh, dry and turgid weights, respectively.

### 2.4 Photosynthetic Pigments and Photosynthetic Activity Analysis

Leaf photosynthetic pigments, gas exchange, chlorophyll fluorescence and CO_2_ response curves were determined at each time point (0, 4, 8 and 12 days after treatment) for each population and treatment in individual young fully expanded leaves (six plants per treatment). Leaf chlorophyll (Chl) content was measured using a portable chlorophyll content (Multi‐Pigment‐Meter MPM‐100, Opti‐ Sciences). Leaf gas exchange, chlorophyll fluorescence and CO_2_ curves were evaluated using a Portable Photosynthesis System (Ciras‐4 equipped with CFM‐4 chlorophyll fluorescence module, PP‐Systems). For combined chlorophyll fluorescence and gas exchange, after a dark‐adaptation period of 30 min, we measured dark and then light‐adapted Chl fluorescence parameters and leaf gas exchange at operational light intensity under cuvette conditions of: 400 μmol mol^−1^ [CO_2_], 200 μmol photon m^−2^ s^−1^ (Photosynthetic Photon Flux Density, PPFD), 60% relative humidity and flow rate 250 μmol s^−1^ and a chamber temperature of 25°C. The measured parameters at operational light intensity included: net photosynthetic rate (A_op_), stomatal conductance (g_s op_), intercellular CO_2_ concentration (C_i op_), maximum photosynthetic efficiency of photosystem II (F_V_/F_M op_), actual photon yield of PSII photochemistry (φPSII_op_), electron transport rate (ETR_op_) and non‐photochemical quenching (NPQ_op_). These measurements were taken always during the same day period (between 09:00 and 13:00). At the end of the 12‐day experiment, after exposing the plants to saturating 1500 μmol m^−2^ s^−1^ PPFD for at least 20 min, we recorded rapid CO_2_ response curves (A/C_i_) at 1500 μmol m^−2^ s^−1^ PPFD, 60% relative humidity, chamber air temperature of 25°C, flow rate 250 μmol s^−1^ and the following external CO_2_ concentrations: 400, 300, 250, 200, 150, 100, 50, 400, 500, 600, 800, 1000 and 1200 μmol mol^−1^. The maximum carboxylation rate of RubisCO (V_cmax_), the maximum rate of electron transport for regeneration of ribulose‐1,5‐bisphosphate (J_max_), the maximum rate of triose‐phosphate utilisation (TPU) and stomatal limitation were calculated following Sharkey (2016). The photosynthetic efficiency was calculated as the A/C_i_ ratio following Salmon et al. (2020).

The experiment ended after 12 days as the non‐metallicolous plants started to show significant declines for all measured parameters due to Tl toxicity. Eighteen additional plants from the metallicolous population (six control plants, six plants 2.5 Tl and six plants 10 μM Tl) were maintained in hydroponics to check the effect of Tl treatment in the long term. Twice a week, Chl fluorescence and stomatal conductance were assessed with a porometer (LI‐600 Porometer/Fluorimeter, Li‐Cor) to monitor plant health. The following parameters were monitored: stomatal conductance (g_sop_), maximum photosynthetic efficiency of photosystem II (F_V_/F_M_), actual photon yield of PSII photochemistry (φPSII_op_) and electron transport rate (ETR_op_). Since the recorded values were stable after two and a half months after the beginning of the treatment, at the weekly full change of the nutrient solution, the Tl concentration was raised to 25 and 100 μM Tl. After 3 weeks, no significant changes were observed; thus, the treatments were raised to 250 and 1000 μM Tl, respectively. The two increasing Tl treatments were identified as the ‘low Tl’ and ‘high Tl’ level treatments. After 3 weeks (i.e., after a total of 4 months of exposure), a significant decline in stomatal conductance in the high Tl treatment was observed. The experiment with the metallicolous plants was concluded, and all parameters were evaluated as described.

### 2.5 Stomatal Density and Stomatal Size

At the end of the experiment, for each of the 12 plants per treatment, young fully expanded leaves were collected and the imprints of both adaxial and abaxial leaf surfaces were taken using the nail polish method as in Pathoumthong et al. (2023). Photos of 10 areas of 0.1 mm^2^ of the same leaf, for a total area of 1 mm^2^, were taken and analysed, using a microscope (model SME‐F8BH binocular microscope) with eyepiece camera (model MD130) and software (AmScope ver. 3.7). In these areas, the cells were counted to assess stomatal density, epidermal cell density, stomatal index (number of stomata to total number of leaf cells) and stomatal size (length and width).

### 2.6 Microscopy Analysis

For optical microscopy analysis, at the end of the exposure time, root and leaf portions of 12 plants for each treatment were fixed in a solution of formaldehyde 10%, ethanol 50%, acetic acid 5% (FAA) for 24 h at 4°C. Samples were then dehydrated progressively by treatment with 70% ethanol for 2 h, 80% ethanol for 2 h, 95% ethanol for 2 h and then twice in absolute ethanol for 4 h, cumulatively. Following the method of Gerrits and Smid (1983), pre‐inclusion was performed first with ethanol and Technovit 7100 historesin in a 1:1 ratio for 2 h, then with a 1:2 ratio for 2 h, and in pure historesin and hardener (I) in a ratio of 1:0.25 for one night. Finally, the specimens were inserted in a polypropylene capsule with the addition of a hardener (II) in a ratio of 1:15 of basic resin. The included samples were cut in 5 μm thick sections with an ultramicrotome and stained with Toluidine Blue O 0.1% (w/v) in phosphate buffer pH 7. Sections were observed with a light microscope (Leitz DM‐RB Fluo) equipped with a digital camera (Nikon Inc.). The images were analysed (with Image J software) to determine the percentage of root central cylinder area, leaf thickness, palisade and spongy mesophyll thickness and percentage of leaf intracellular spaces.

Transmission electron microscopy (TEM) images were obtained from control and 10 μM Tl‐treated plants. Portions of leaf samples from 12 plants for each treatment were collected and immediately fixed in 1.25% (v/v) glutaraldehyde in 0.1 M phosphate buffer (pH 7.2), stored at 4°C for 24 h and subsequently fixed in 1% OsO_4_ in 0.1 M phosphate buffer (pH 7.2). After an ethanol series dehydration, samples were embedded in Spurr epoxy resin (Spurr 1969). Cross and longitudinal sections of both roots and leaves were cut using a Reichert‐Jung ULTRACUT ultra microtome with a diamond knife. The 70 nm thick sections, placed on a copper grid, were subsequently stained with uranyl acetate (Gibbons and Grimstone 1960) and lead citrate (Reynolds 1963). The observations were performed with a JEM 1010 electron microscope (Jeol) at 80 kV.

### 2.7 Statistical Analysis

A two‐way ANOVA (statistical significance when *p* < 0.05) analysis was performed to evaluate the effect of Tl treatment on the two accessions (non‐metallicolous and metallicolous plants) and their interaction. Post hoc comparisons were carried out using the HSD‐Tukey test using GraphPad Prism 7 (GraphPad Software) after checking data normality distribution (assessed with the Shapiro–Wilk test). For growth parameters measured at different time points, a one‐way ANOVA (*p* < 0.05) was used to assess the differences between populations treated with the same Tl concentration at the same time points.

## 3 Results

### 3.1 Growth and Tl Accumulation Over Time in Metallicolous and Non‐Metallicolous Plants

**FIGURE 1:**
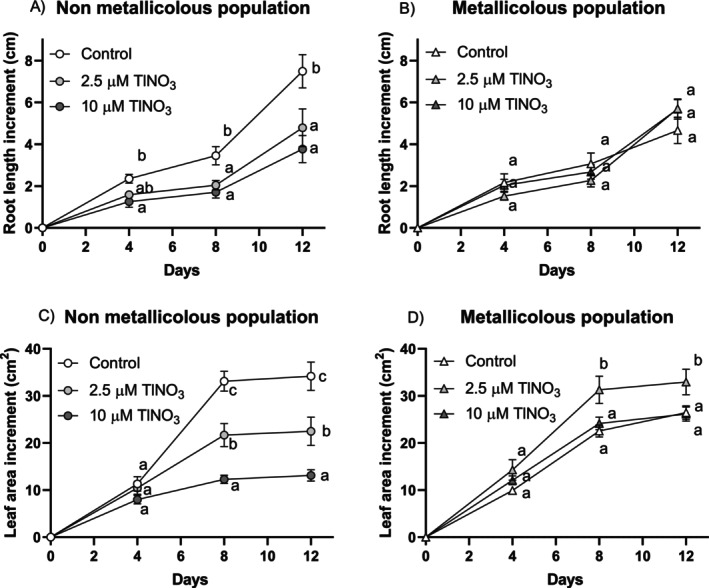
Increment in: (A, B) root length and (C, D) leaf area of the non‐metallicolous and the metallicolous populations of *S. latifolia* exposed to TlNO_3_ for 12 days. Increment was calculated by subtracting values at the beginning of the treatment from values at the end of the treatment. Values are means ± SE of 12 replicates. Letters close to the data points indicate the significant differences among samples at each single exposure time according to the Tukey's test (at least *p* < 0.05).

**FIGURE 2:**
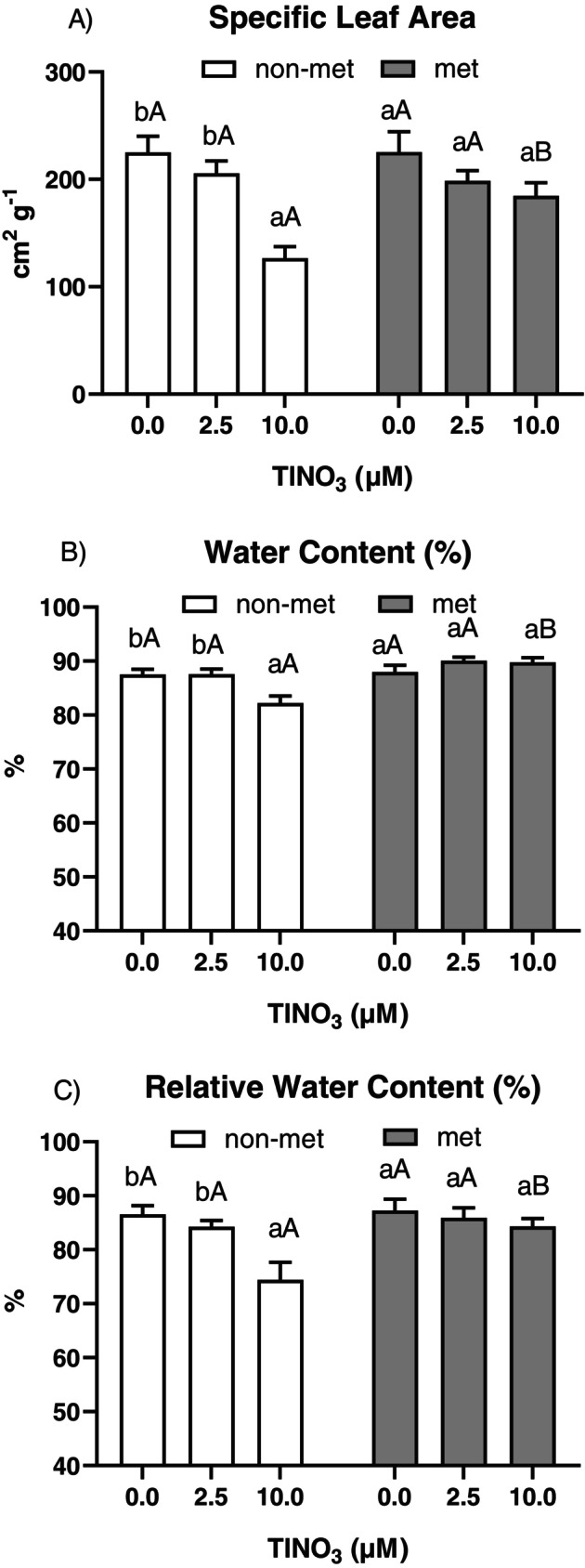
(A) Specific leaf area, (B) leaf water content and (C) leaf relative water content of the non‐metallicolous and the metallicolous populations of *S. latifolia* exposed to Tl for 12 days. Values are means ± SE of 12 replicates. Letters indicate the significant differences among samples according to the Tukey's test (at least *p* < 0.05), capital for inter‐population comparison and lower for intra‐population comparison.

**FIGURE 3:**
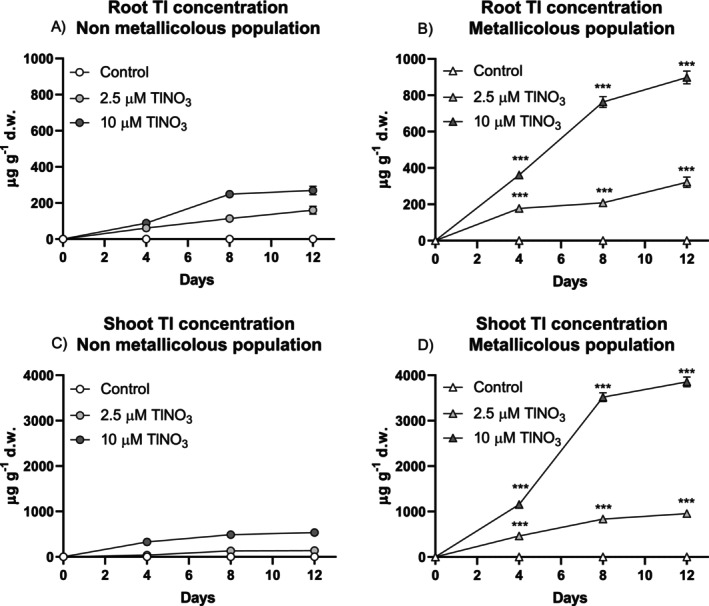
Thallium concentration in: (A, B) roots and (C, D) shoots of the non‐metallicolous and the metallicolous populations of *S. latifolia* exposed to TlNO_3_ for 12 days. Values are means ± SE of 12 replicates. Asterisks close to the data points of the MET population indicate the significant differences between the same treatment at the same time in respect to the NON‐MET population according to the Tukey's test (***p < 0.001).

Before assessing the time‐dependent effect of Tl on photosynthetic parameters, growth and Tl accumulation were monitored (Figure 1). For both root length and leaf area increments, a significant interaction treatment versus time was found (Table S1) only in the non‐metallicolous plants, where Tl exposure significantly reduced growth compared to controls. By contrast, metallicolous plants showed no significant growth reduction in response to Tl and, at the lowest concentration, Tl even promoted an increase in leaf area increment compared to controls. There were significant differences for the interaction population versus treatment (Table S2) for the monitored leaf traits at the end of the treatment (Figure 2). In the non‐metallicolous plants, SLA (specific leaf area), WC (water content) and RWC (relative water content) were significantly lower at the highest Tl treatment compared to controls. On the other hand, all parameters in the metallicolous plants were not affected by Tl exposure. Thallium concentrations in the roots and shoots of non‐metallicolous and metallicolous plants increased with increasing metal concentrations in the root zone and exposure time (Figure 3). Thallium concentrations in shoots were much higher than those in the roots for both populations. The metallicolous plants had significantly higher root and shoot Tl concentrations compared to the non‐metallicolous plants at both Tl exposure levels, with a significant interaction treatment versus time (Table S3).

### 3.2 Photosynthetic Parameters in Presence of Thallium

**FIGURE 4:**
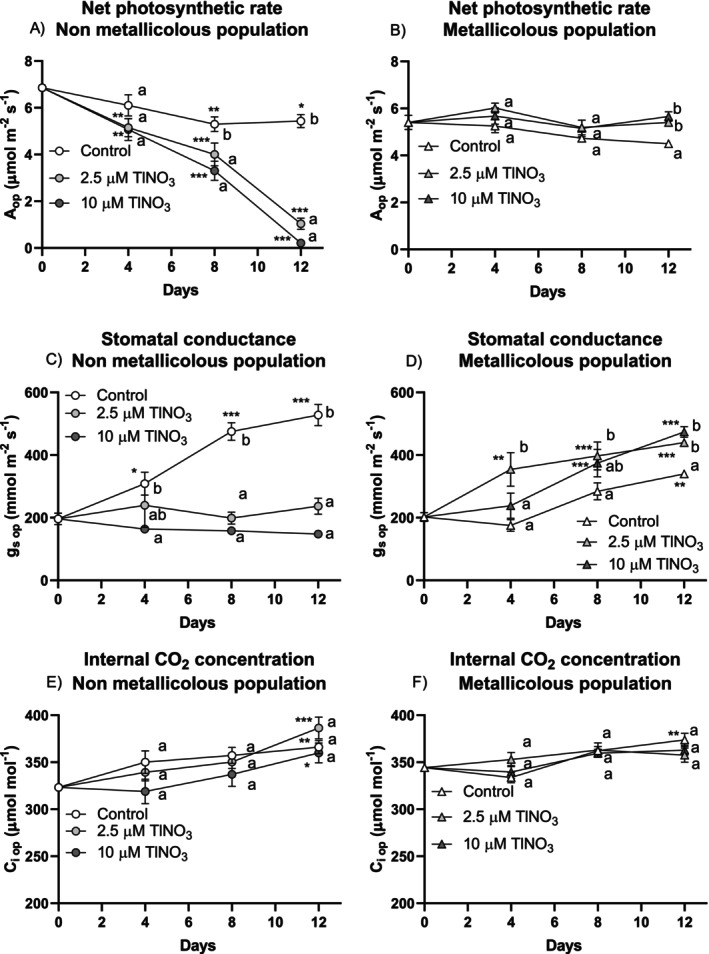
Time‐dependent changes in gas exchange parameters in the non‐metallicolous and the metallicolous populations of *S. latifolia* exposed to Tl for 12 days. (A, B) net photosynthetic rate A_op_, (C, D) stomatal conductance g_s op_, (E, F) internal CO_2_ concentration C_i op_. Values are means ± SE of 6 replicates. According to the Tukey's test (at least *p* < 0.05), asterisks close to the data points indicate significant differences within the same treatment with respect to the beginning of the exposure (*p < 0.05; **p < 0.01; ***p < 0.001), whereas letters indicate the significant differences among samples at each single exposure time.

Gas exchange measurements, conducted under the operational conditions of the growth chamber, revealed distinct responses between the two populations to the increasing Tl in the substrate. In Tl‐treated non‐metallicolous plants, A_op_ (net photosynthetic rate) declined significantly after 8 days of exposure with a significant interaction treatment versus time (Figure 4A; Table S4A). By contrast, in metallicolous plants, not only Tl presence in the root‐zone did not affect A_op_ compared to controls, but it also increased the values of this parameter compared to controls at the end of the experiment (Figure 4B). Similar results were obtained for g_s op_ (stomatal conductance), with a general decrease in the non‐metallicolous population in the presence of Tl and an increase, significant from the first time point, in the metallicolous population compared to controls (Figure 4C,D, Table S4C,D). In both groups of plants, Tl in the root‐zone did not affect C_i op_ (internal CO_2_ concentration) across treatments, aside from a general increase over time, significant at the end of the 12 days treatment in non‐metallicolous plants and, in metallicolous plants, only under control conditions (Figure 4E,F; Table S4E,F).

**FIGURE 5:**
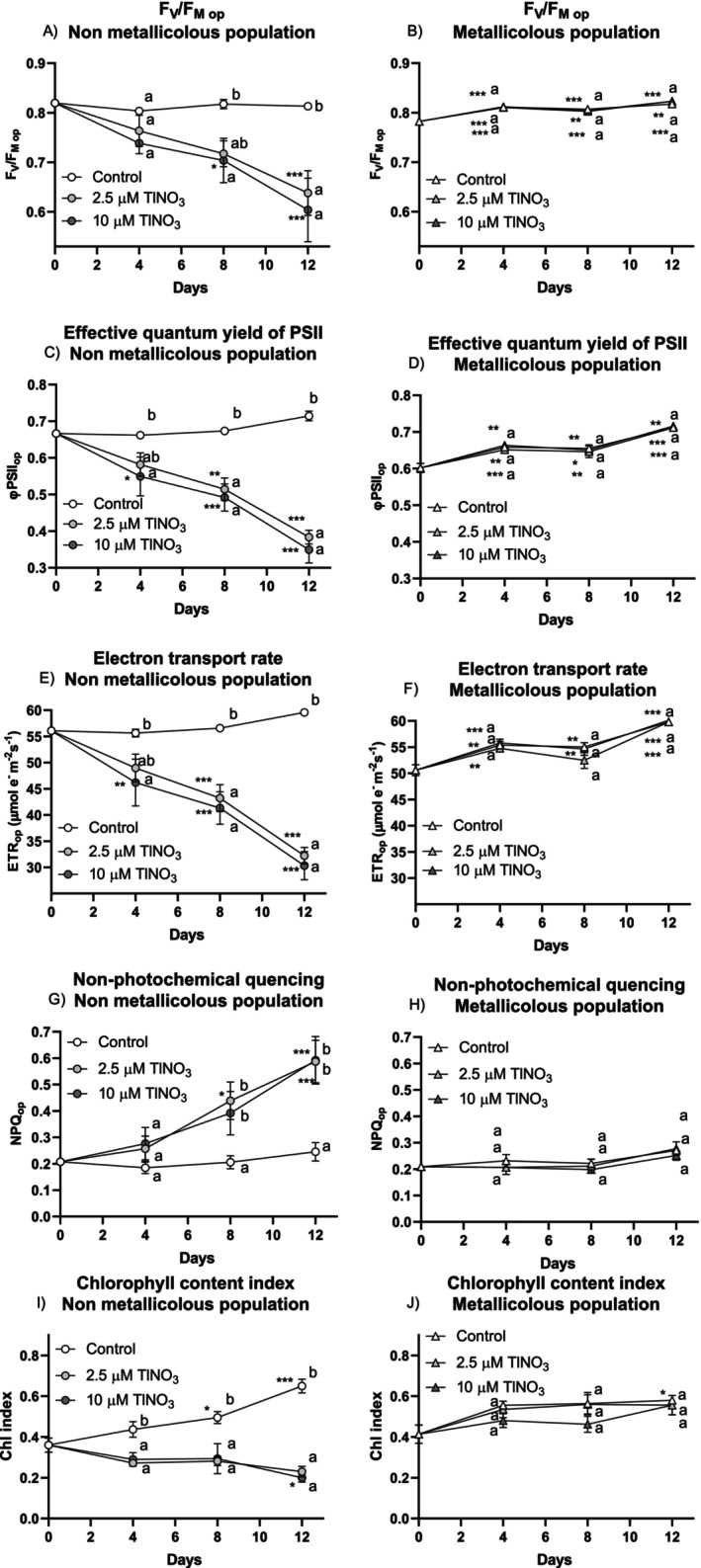
Time‐dependent changes in fluorescence parameters in the non‐metallicolous and the metallicolous populations of *S. latifolia* exposed to Tl for 12 days. (A, B) F_V_/F_M_, (C, D) φPSII, (E, F) ETR and (G, H) NPQ. (I, J) Chlorophyll content index of the same samples. Values are means ± SE of 6 replicates. According to the Tukey's test (at least *p* < 0.05), asterisks close to the data points indicate significant differences within the same treatment in respect to the beginning of the exposure (*p < 0.05; **p < 0.01; ***p < 0.001), whereas letters indicate the significant differences among samples at each single exposure time.

Similarly to leaf gas exchange, time‐dependent changes in leaf chlorophyll fluorescence parameters measured under operational conditions had a significantly different trend between the non‐metallicolous and metallicolous plants in the presence of Tl (Figure 5; Table S5). In the non‐metallicolous plants, F_V_/F_M_ (maximum quantum yield of PSII), φPSII_op_ (effective quantum yield of PSII) and ETR_op_ (electron transport rate) had a significant decline, starting from the first time point, compared to the control. In the metallicolous plants, these parameters were not affected by Tl and had a general increase over time. Accordingly, NPQ values increased in a Tl‐dependent manner in the non‐metallicolous plants while remaining constant in the metallicolous plants. The leaf chlorophyll content index (Figure 5I,J) was not affected by Tl in the metallicolous plants for the entire duration of the experiment, whilst in the non‐metallicolous plants, there was a Tl‐induced reduction compared to the control at each time point (Table S5I,J).

**FIGURE 6:**
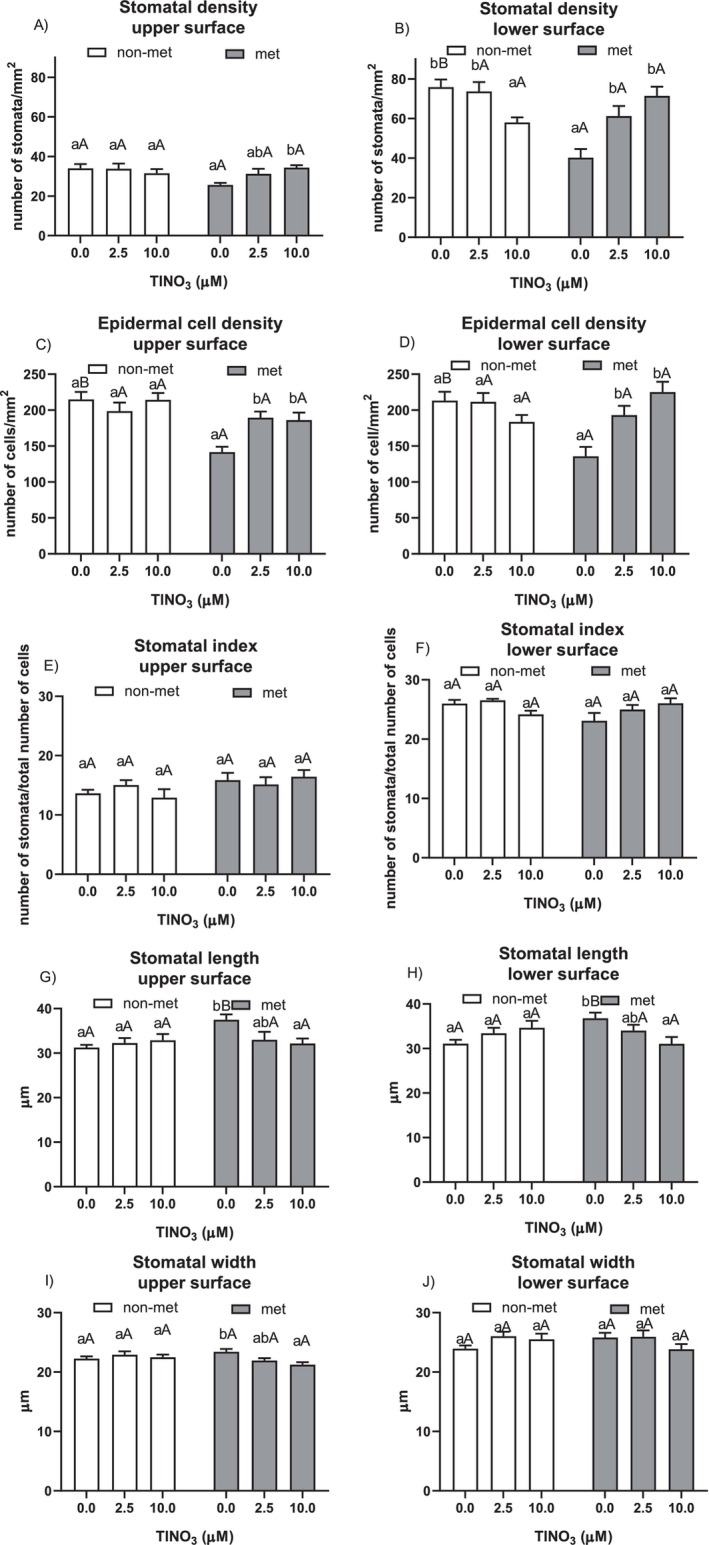
(A) stomatal density upper surface, (B) stomatal density lower surface, (C) epidermal cell density upper surface, (D) epidermal cell density lower surface, (E) stomatal index upper surface, (F) stomatal index lower surface, (G) stomatal length upper surface, (H) stomatal length lower surface, (I) stomatal width upper surface and (J) stomatal width lower surface of the non‐metallicolous and the metallicolous populations of *S. latifolia* exposed to Tl for 12 days. Values are means ± SE of 12 replicates. Letters indicate the significant differences among samples according to the Tukey's test (at least *p* < 0.05), capital for inter‐population comparison and lower for intra‐population comparison.

Stomatal density and pore size were measured in both populations at the end of the 12 days treatment (Figure 6). In the non‐metallicolous plants, the Tl treatments led to a significant decrease in stomatal density in the lower leaf surface, but in the metallicolous plants, the presence of the metal in the root zone induced a significant increase in stomatal density and epidermal cell density in both leaf surfaces. Nevertheless, in metallicolous plants, the stomatal index and pore size remained constant, except for a reduced stomatal length in the lower leaf surface in the highest Tl treatment. The interaction population versus treatment was consistently significant, except for the stomatal width of the lower surface (Table S6).

**FIGURE 7:**
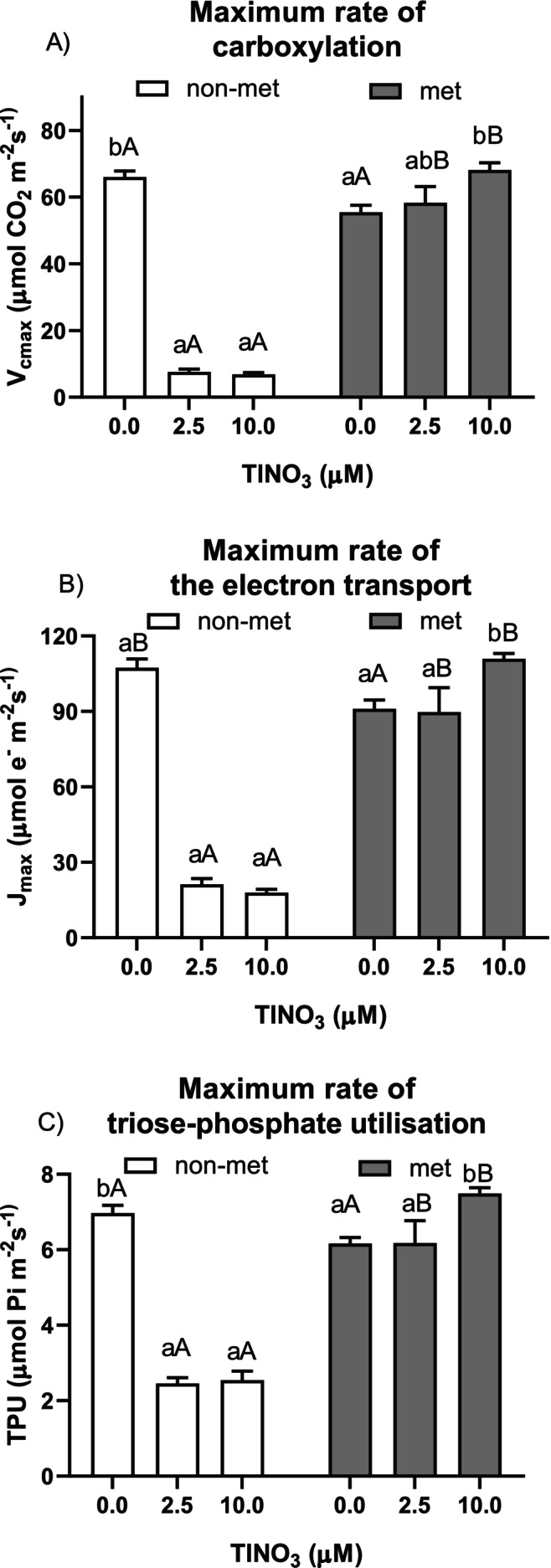
Photosynthetic biochemical parameters: (A) V_cmax_, (B) J_max_ and (C) TPU of the non‐metallicolous and the metallicolous populations of *S. latifolia* exposed to TlNO_3_ for 12 days. Values are means ± SE of 6 replicates. Letters indicate the significant differences among samples according to the Tukey's test (at least *p* < 0.05), capital for inter‐population comparison and lower for intra‐population comparison.

**FIGURE 8:**
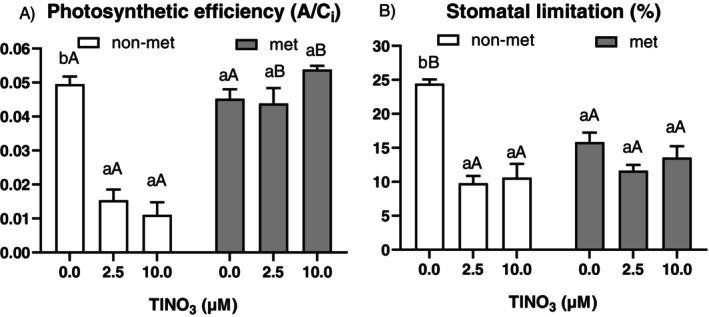
Photosynthetic limitations: (A) Photosynthetic efficiency and (B) Stomatal limitation of the non‐metallicolous and the metallicolous populations of *S. latifolia* exposed to Tl for 12 days. Values are means ± SE of 6 replicates. Letters indicate the significant differences among samples according to the Tukey's test (at least *p* < 0.05), capital for inter‐population comparison and lower for intra‐population comparison.

Rapid A/C_i_ curves were recorded to calculate the key biochemical factors apparent V_cmax_ (maximum carboxylation rate of Rubisco), apparent J_max_ (maximum rate of the electron transport) and apparent TPU (maximum rate of triose‐phosphate utilisation), as reported in Figure 7. There was a significant interaction between population and treatment (Table S7), revealing a Tl‐induced reduction in all parameters only in the non‐metallicolous accession, while in the metallicolous population, there was a significant increase at the highest metal concentration compared to controls. Estimated Tl‐induced limitations to photosynthesis indicated that, after 12 days of treatment, in non‐metallicolous plants, there was a marked decline in both photosynthetic efficiency and stomatal limitation at the 2.5 and 10 μM Tl treatment levels. On the other hand, no significant differences were observed for metallicolous plants (Figure 8).

In the metallicolous population grown for 4 months in the presence of the metal, the lower Tl treatment did not have any significant effects for almost all the measured parameters compared to the control; whereas, the highest Tl treatment reduced plant growth and gas exchange, with a significant decline in most of the parameters related to biochemical photosynthetic capacity (Table S9). In contrast, the parameters derived from chlorophyll fluorescence analysis were not affected by the presence of Tl.

### 3.3 Anatomical and Ultrastructural Analysis

**FIGURE 9:**
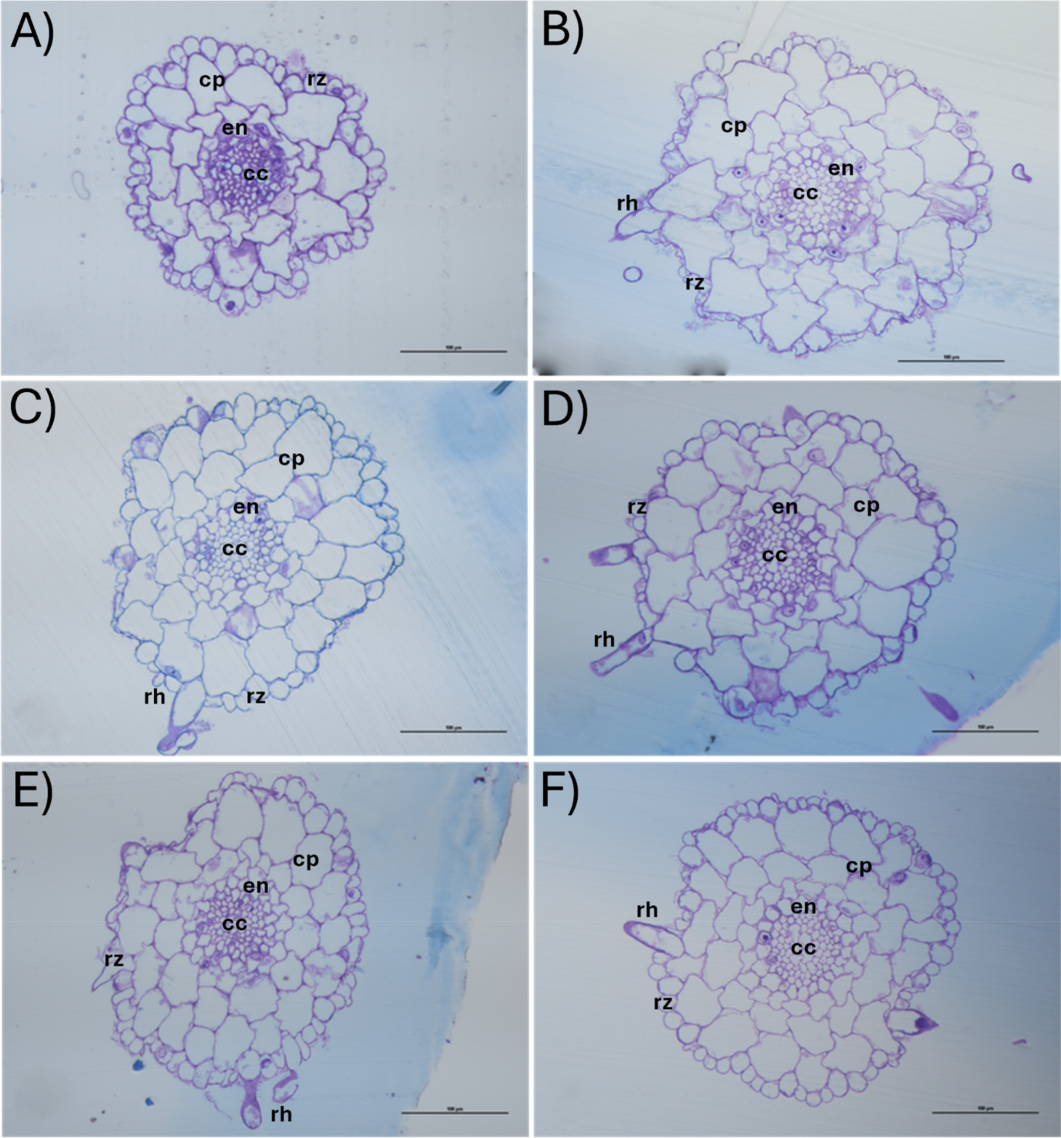
Cross sections close to the root apex (less than 1 mm) of the non‐metallicolous and the metallicolous populations of *S. latifolia* exposed to Tl for 12 days. (A) control non‐metallicolous, (B) control metallicolous, (C) 2.5 μM Tl non‐metallicolous, (D) 2.5 μM Tl metallicolous, (E) 10 μM Tl non‐metallicolous and (F) 10 μM Tl metallicolous. cc, central cylinder; cp, cortical parenchyma; en, endodermis; rh, root hair; rz, rhizodermis.

**FIGURE 10:**
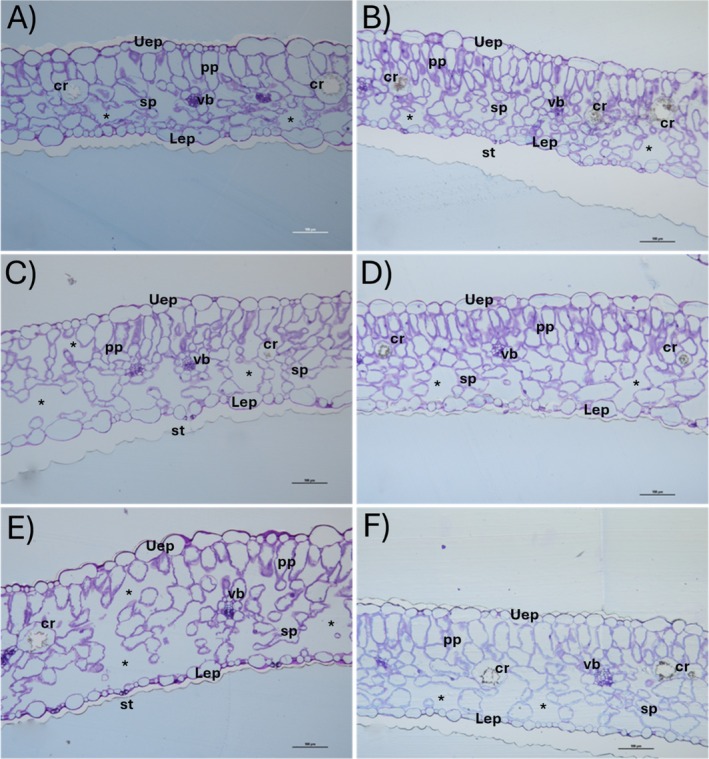
Leaf cross sections of the non‐metallicolous and the metallicolous populations of *S. latifolia* exposed to Tl for 12 days. (A) control non‐metallicolous, (B) control metallicolous, (C) 2.5 μM Tl non‐metallicolous, (D) 2.5 μM Tl metallicolous, (E) 10 μM Tl non‐metallicolous and (F) 10 μM Tl metallicolous. *, intercellular spaces; cr, crystal; Lep, lower epidermis; pp., palisade parenchyma; sp., spongy parenchyma; st, stomata; Uep, Upper epidermis; vb, vascular bundle.

The general organisation of the maturing root zone of the non‐metallicolous and metallicolous plants did not differ in the control samples. The only exception was a more pronounced xylem differentiation in the non‐metallicolous plants (Figure 9A) compared to metallicolous ones (Figure 9B), as indicated by the presence of toluidine‐reactive blue–green elements in the central cylinder. In the presence of Tl, while the roots of metallicolous plants had a well‐defined anatomy (Figure 9B,D,F), the roots of the non‐metallicolous plants (Figure 9A,C,E) became progressively more disorganised, losing the normal structure of approximately three concentric layers of parenchyma cells outside the endodermis, while the endodermis itself seemed discontinuous at the highest Tl concentration. A similar trend was observed in leaf cross sections, with increasing disorganisation of both palisade and spongy mesophyll in the Tl‐exposed non‐metallicolous plants (Figure 10A,C,E) and negligible effects in the contrasting group of metallicolous plants (Figure 10B,D,F). The leaf anatomy of the non‐metallicolous plants with the Tl treatment had very large intercellular spaces in the palisade and, especially, in the spongy parenchyma (Figure 10C,E).

**FIGURE 11:**
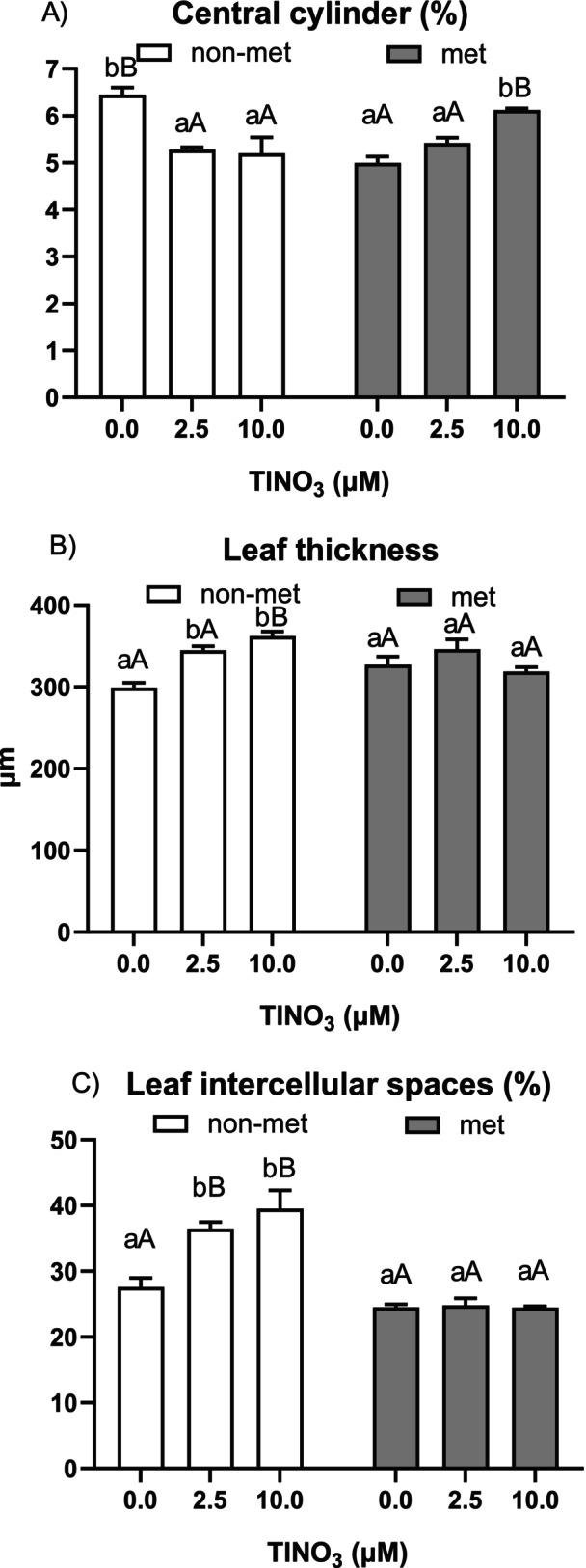
(A) percentage of central cylinder area in cross sections close to the root apex (less than 1 mm), (B) leaf thickness and (C) percentage of leaf intercellular space area of the non‐metallicolous and the metallicolous populations of *S. latifolia* exposed to Tl for 12 days. Values are means ± SE of 12 replicates. Letters indicate the significant differences among samples according to the Tukey's test (at least *p* < 0.05), capital for inter‐population comparison and lower for intra‐population comparison.

The analysis of the root and leaf sections showed a significant effect for most of the quantified features (Figures 11 and S2; Table S10). Upon Tl exposure, the percentage of central cylinder area significantly decreased in non‐metallicolous plants and increased in metallicolous plants, with a significant interaction between population and treatment. Regarding leaves, non‐metallicolous plants had increasing values in leaf thickness at increasing Tl concentrations, along with a concomitant increase in both palisade and spongy mesophyll thickness that resulted in a stable proportion between each. In the plants, the percentage of intercellular spaces increased as well following Tl treatment, whereas in the metallicolous samples, all of the parameters did not change upon Tl treatment.

**FIGURE 12:**
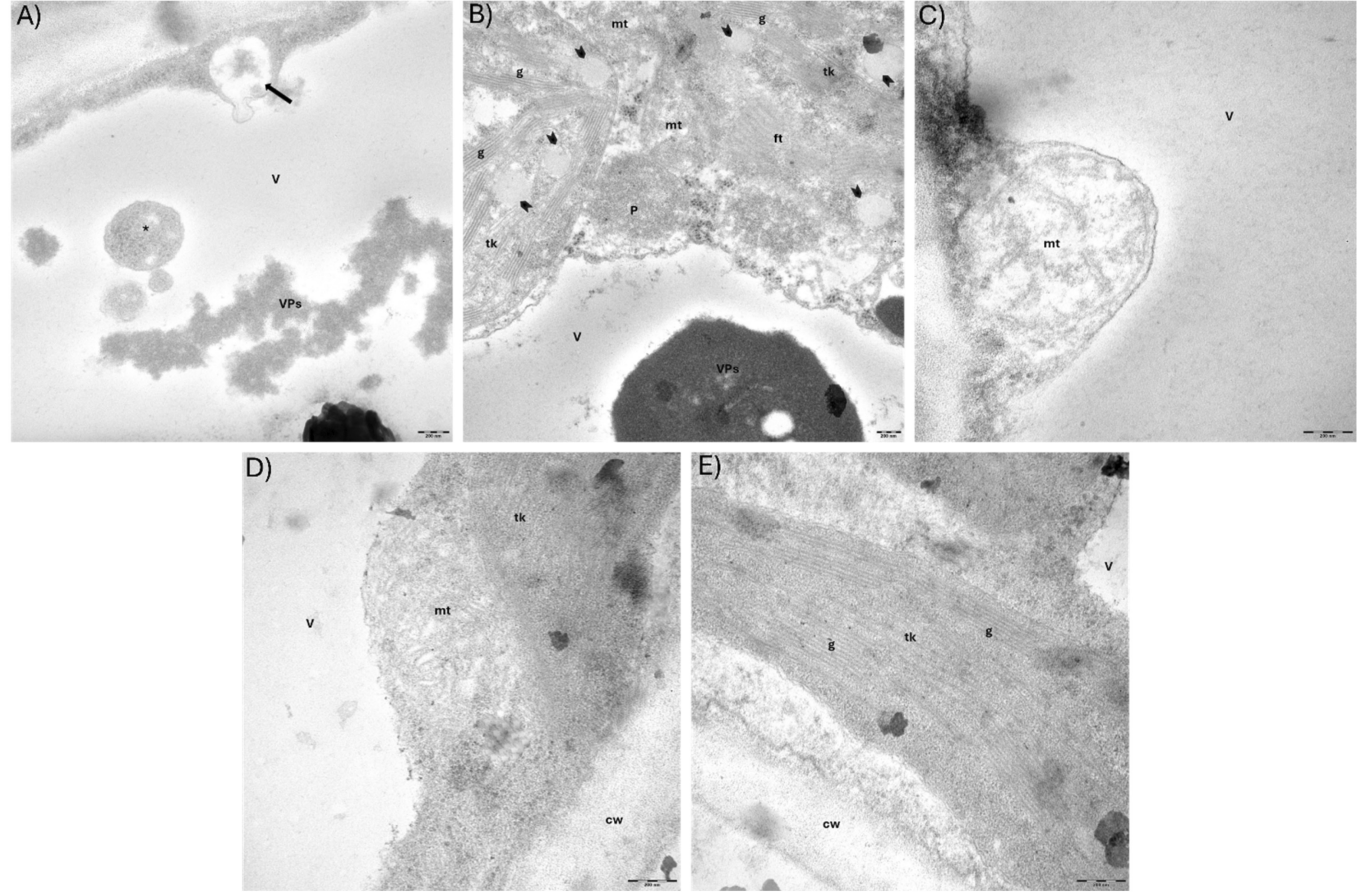
Mesophyll cells of the non‐metallicolous (A–C) and metallicolous (D, E) population exposed to 10 μM Tl. (A) Electron‐dense precipitates in the vacuole together with cytoplasm portions (asterisk) and a vesicle containing electron‐dense material crossing the tonoplast (arrow). (B) Chloroplasts with low electron‐dense stroma and large plastoglobuli (arrow heads); electron‐dense precipitates can be seen in the vacuole. (C) Mitochondrion with few cristae and low electron density. (D) Mitochondrion with numerous cristae and with higher electron density compared to that in (C). (E) Chloroplasts with electron‐dense stroma and no plastoglobuli. cw, cell wall; ft., fused thylakoids; g, grana; mt, mitochondria; tk, thylakoids; V, vacuoles; VPs, precipitates. Scale bar: 200 nm.

As for the ultrastructural analysis, in control conditions (Figure S3), the mesophyll cells from both populations showed similar features, with well‐organised chloroplasts, containing large starch granules and few or no plastoglobuli, and electron‐dense mitochondria with numerous cristae. In the cytoplasm, abundant ribosomes were present and the vacuoles were transparent. In the presence of 10 μM Tl, the mesophyll cells of the non‐metallicolous population showed electron‐dense precipitates in the vacuole (Figure 12A,B). The chloroplasts displayed rarefied stroma with several large plastoglobuli, around 200–250 nm in diameter and thylakoids that frequently seemed fused together (Figure 12B), while the mitochondria showed low electron density and few cristae (Figure 12C). Many portions of cytoplasm could be observed in the vacuole (Figures 12A and S4A) and the vacuolar electron‐dense material was very similar to electron‐dense bodies formed in the cytoplasm between chloroplast and tonoplast (Figure S4B). In comparison, the mesophyll cells of the metallicolous population showed electron‐dense mitochondria with many cristae (Figure 12D) and chloroplasts with electron‐dense stroma and no plastoglobuli (Figure 12E). In addition, the cytoplasm showed a higher number of ribosomes with respect to the non‐metallicolous population (Figures 12 and S4A,B).

## 4 Discussion

### 4.1 Thallium Requirement for Optimal Growth in the Hyperaccumulating Population

All available data confirm that *S. latifolia* is a facultative Tl hyperaccumulator, with Tl having a remarkable physiological effect on this species. In other recent studies, a growth‐promoting effect upon Tl exposure has also been found in the facultative Tl hyperaccumulator *Biscutella laevigata* (Salinitro et al. 2024). Stimulatory effects have also been reported for plant species hyperaccumulating Cd (Roosens et al. 2003; Deng et al. 2007), As (Ma et al. 2001) and Se (Statwick et al. 2016). The general assumption is that hyperaccumulators have a higher basal requirement for the hyperaccumulated metal/metalloid (Pollard et al. 2002; Manara et al. 2020), but a convincing mechanistic explanation has thus far not been provided for this effect. In the case of Zn or Ni, the stimulatory effect at low doses has been attributed to the strong expression of constitutive sequestration mechanisms that deplete their cytosolic concentrations (Talke et al. 2006; Hanikenne et al. 2008; Bettarini et al. 2021; Colzi et al. 2023). Under the experimental conditions of this study, the metallicolous population showed the first signs of Tl‐imposed growth reduction only when the Tl concentrations were raised to extremely high values, with foliar accumulation reaching 15,000 μg Tl g^−1^. The high tolerance of metallicolous plants resulted in a stable SLA and tissue hydration state at the end of the exposure time. A similar stability has been observed for Ni hyperaccumulators under Ni treatment (Scartazza et al. 2022), where it has been attributed to the osmotic adjustment processes and preferential accumulation of the metal in the leaf epidermis (Whiting et al. 2003; McNear et al. 2010). In contrast, the Tl treatment reduced SLA in the non‐metallicolous plants, as frequently reported for metal‐stressed plants (Yadav et al. 2021), probably due to the metal‐imposed water stress (Barceló and Poschenrieder 2008). This hypothesis is supported by the observed decrease in water content and relative water contents with high Tl exposure. The SLA reduction was also evident from the analysis of leaf cross sections, where Tl induced an increase in the thickness of both palisade and spongy mesophyll only in non‐metallicolous plants. A similar response has also been reported in an experiment on Tl toxicity in *Sinapis alba* (Mazur et al. 2016). Despite these changes in the non‐metallicolous plants, biometric data indicated that, for most of the applied combinations of Tl concentrations and exposure times, all plants, including those from the non‐metallicolous population, were still growing and, therefore, metabolically active to display physiologically relevant responses to Tl‐imposed stress.

### 4.2 CO_2_ Photosynthetic Assimilation Increased in the Presence of Tl in the Hyperaccumulating Population

In operational light conditions, non‐metallicolous plants showed the typical Tl‐induced reduction of A_op_, associated with partial stomatal closure, at almost all the concentrations and times (see, e.g., Domínguez et al. 2011). This reduced CO_2_ fixation likely contributes to the observed growth reduction in the presence of Tl. In contrast, after 12 days of treatment, both stomatal opening and photosynthetic rates improved in the Tl‐treated metallicolous plants, reaching values similar to those in control non‐metallicolous plants. This coincided with Tl tissue concentrations far exceeding the hyperaccumulation threshold of 100 μg Tl g^−1^. Interestingly, the first observed Tl‐induced stimulatory response in the metallicolous plants was an improved stomatal opening that probably led to the subsequent rise in net photosynthesis. Overall, in both non‐metallicolous and metallicolous plants, C_i op_ remained constant throughout the duration of the experiments and Tl exposure. This C_i op_ stability in the metallicolous population resulted from the parallel increase in both A_op_ and g_s op_, while in the non‐metallicolous plants it was associated with a simultaneous decline in both CO_2_ diffusion within the leaf and efficiency of carbon assimilation. While the improved gas exchange may partially explain the enhanced growth of metallicolous plants at the lowest Tl exposure, the absence of this phenomenon for the highest Tl exposure could derive from the increasing cost of Tl detoxification mechanisms.

A metal‐induced stimulation of stomatal opening has been reported in Ni hyperaccumulators of the genus *Odontarrhena* (Scartazza et al. 2022). In these species, this is considered to be a mechanism to increase Ni accumulation level in shoots as long as Ni is excluded from guard cells to preserve stomatal function (Psaras 2000). Given that in *S. latifolia* stomata have also been found to remain Tl‐free compared to the surrounding epidermis (Corzo Remigio, Nkrumah, et al. 2022), a similar mechanism for stomata protection through exclusion could be hypothesised for Tl. This is particularly relevant considering that, in sensitive plants, Tl has been found to induce stomatal closure through interference of the K movement between guard and subsidiary cells (Pallaghy 1972). Accordingly, the uneven Tl distribution observed across the leaf epidermal cells in *S. latifolia* , with Tl accumulating preferentially at the base of the trichomes, has already been proposed as a protection mechanism for photosynthetically active mesophyll cells (Corzo Remigio, Nkrumah, et al. 2022).

The stimulatory effects of Tl on stomatal opening in the metallicolous plants could derive from Tl‐induced morpho‐physiological changes to boost water uptake and thus Tl translocation in the shoots. Supporting this hypothesis, the increased proportion of the central cylinder area in the apical zone of the roots of Tl‐treated metallicolous plants, to values similar to those in controls in non‐metallicolous ones, would allow for higher xylem hydraulic conductivities, thus explaining the observed increases in stomatal conductance rates. In non‐metallicolous plants, the opposite trend was found, with Tl inducing a reduction in the area covered by the vascular cylinder and impairing xylem development; both are likely to have affected long‐distance water transport, thus leading to the observed Tl‐imposed decrease in g_s_. Similar damages at the root level have been previously reported as a result of trace metal excess (Yadav et al. 2021). Furthermore, the analysis of stomatal traits revealed that the non‐metallicolous and metallicolous plants had contrasting features. Indeed, the greater g_s op_ in Tl‐treated plants was clearly associated with higher stomatal densities on both the upper and lower leaf surfaces, with values similar to non‐metallicolous plants in control conditions. The concurrent increase in epidermal cell numbers, along with the stable stomatal index and lower length, indicates that the metallicolous plants adjusted their stomatal densities through an increase in the total number of epidermal cells rather than an increase in stomatal differentiation rate. In contrast, in non‐metallicolous plants, Tl‐induced changes resulted only in a reduced stomatal density in the lower leaf surface, as, for example, reported for Zn excess (Shi and Cai 2009). While these changes, coupled with the increase in lamina thickness, could have potentially reduced water uptake and Tl translocation, they were also likely a direct consequence of Tl‐induced tissue damage and water stress. Confirming this, leaf cross sections revealed several Tl‐imposed damages in non‐metallicolous plants, with substantial disorganisation of both the parenchyma with increasing intracellular spaces. Such structural disruptions are commonly reported as indicators of trace metal stress (Yadav et al. 2021) and likely contributed to the observed reductions in all measured photosynthetic parameters. Similarly, in the non‐metallicolous population, Tl‐imposed damages to the mesophyll cells were also evidenced at the ultrastructural level. Chloroplasts appeared with anomalously developed grana, rarefied stroma and plastoglobuli, as partly reported by the only available report on Tl‐induced ultrastructural changes and associated with declines in the fluorescence parameters (Mazur et al. 2016). Here, clear signs of damage were shown by both the photosynthetic membranes and the stroma, partly explaining the Tl‐induced impairments to both phases of photosynthesis. Furthermore, the formation of plastoglobuli, already known as a symptom of stress for other metals such as nickel or copper (see, e.g., Kukkola et al. 2000; Molas 2002), was also shown for Tl excess. The quantity and size of plastoglobuli are known to increase in response to many types of environmental stresses, including trace metals (Arzac et al. 2022), since they derive from lipid remodelling and antioxidant and enzyme accumulation (Bréhélin et al. 2007; Rottet et al. 2015). Another clear sign of Tl toxicity in the non‐metallicolous population was the disappearance of cytoplasmic ribosomes and the autophagy of numerous ribosome‐containing portions of cytoplasm. The same effects were observed in human cells and attributed to Tl occupying certain K‐binding sites on the 60S ribosomal subunit and inactivating ribosomal function (Chou and Lo 2019) and to the induction of autophagy as a cytoprotective mechanism (Salvatierra‐Fréchou and Verstraeten 2024). The interference between Tl and K could have been responsible also for the altered cristae in mitochondria from Tl‐treated non‐metallicolous plants, as K homeostasis is well known to be fundamental for the correct development and functioning of such organelles (Ježek et al. 2023).

### 4.3 Thallium Accumulation Did Not Affect Light Reactions in the Hyperaccumulating Population

In the non‐metallicolous plants, the metal‐induced decrease in CO_2_ assimilation rate was partly explained by the impaired photochemical reactions, since Chl content, PSII photochemistry and electron transport rate were all negatively affected. These changes were associated with an increase in NPQ, suggesting a greater need to dissipate excess excitation energy by non‐photochemical quenching in non‐metallicolous plants. A similar response has also been reported in other species and mainly attributed to Tl‐mediated oxidative stress and protein denaturation through —SH binding (Mazur et al. 2016; Espinosa et al. 2023). By contrast, in the metallicolous population, the light reactions remained unchanged despite significantly higher Tl concentration in leaf tissues compared to non‐metallicolous plants. This serves as strong evidence supporting that metal hyperaccumulation mechanisms protect the photosynthetic machinery, as previously hypothesised by the F_V_/F_M_ test (Regini et al. 2024). Under the experimental conditions used in this study, there was no evidence that Tl increased PSII photochemistry and electron transport rate. This contrasts with findings for Ni hyperaccumulators from the genus *Odontarrhena*, where high Ni concentrations, far exceeding those required for the simple improvement of cytosolic Ni nutrition in the presence of Ni hyperaccumulation mechanisms, were required for optimal light reactions (Scartazza et al. 2022). The absence of a Tl‐mediated amelioration of the photochemical reactions could be attributed to the fact that this element may not have a physiological role in the plant, at least for the processes considered here. Instead, Tl accumulation may have an anti‐herbivory role, in line with the ‘Elemental Defence’ hypothesis (Boyd 2007).

### 4.4 Thallium Improved Maximal Photosynthetic Activity in the Hyperaccumulating Population

As mentioned above, the Tl‐imposed limitations to photosynthesis in sensitive plants have already been reported in other species and attributed to restrictions in both gas diffusional and photochemical processes. Concerning the biochemical limitations, we report for the first time that Tl negatively affected carboxylation activity and Calvin cycle functionality, as clearly shown by the reduction in V_cmax_, J_max_ and TPU in Tl‐treated non‐metallicolous plants. For other metals, similar declines in these parameters have been recognised as general indicators of metal toxicity (see, e.g., Borghi et al. 2008), leading to ATP and NADPH accumulation, which in turn trigger feedback inhibition of the PSII electron transport (Krupa and Baszynski 1995). As for Tl, the decrease in Rubisco activity could also depend on interference with its folding process, since the GroEL chaperonine is known to have an absolute requirement for K (Gruber and Horovitz 2016) and a Tl replacement cannot be excluded. Interestingly, in the metallicolous plants, not only the Rubisco and Calvin cycle components remained functional under Tl exposure, but photosynthetic biochemistry also improved at 10 μM Tl, with V_cmax_, J_max_ and TPU values significantly higher than those in control plants, reaching values measured in control non‐metallicolous plants. However, given that there was no parallel increase in photosynthetic efficiency (A/C_i_) in the presence of Tl, this improvement was likely driven only by a higher CO_2_ supply to the Rubisco carboxylation centres, which increased Calvin cycle efficiency and, indirectly, the electron transport rate through NADPH and ATP consumption. It could be argued that the presence of Tl induced structural changes within the leaf, which, in addition to or correlated with the increased stomatal density, improved water transport and consequent metal accumulation, thereby collaterally promoting CO_2_ trafficking towards chloroplasts. The stable stomatal limitations along Tl treatments further supported the hypothesis of an increased CO_2_ diffusion from the atmosphere to the stroma and thus CO_2_ fixation, which would explain the higher V_cmax_, J_max_ and TPU values as mentioned above. The first signs of Tl toxicity in the metallicolous plants, that is, significant reductions in photosynthetic rates in operational conditions, linked with both diffusional and biochemical limitations, appeared only in the long term with very high and unrealistic, Tl concentrations in the root zone. The light reactions, by contrast, were not affected even at so remarkably high metal accumulation in the leaves (~15,000 μg Tl g^−1^), whereas they have always been reported as a typical target of Tl toxicity in non‐accumulators at far lower tissue element concentrations (Mazur et al. 2016; Espinosa et al. 2023). Therefore, the hyperaccumulation mechanisms, whatever they are, appeared to protect the photosynthetic machinery, especially at the level of the light reactions, since the metallicolous population did not exhibit the same contemporary pattern of Tl‐imposed limitations, just shifted in the metal concentrations in which it occurred, as the non‐metallicolous population.

**FIGURE 13:**
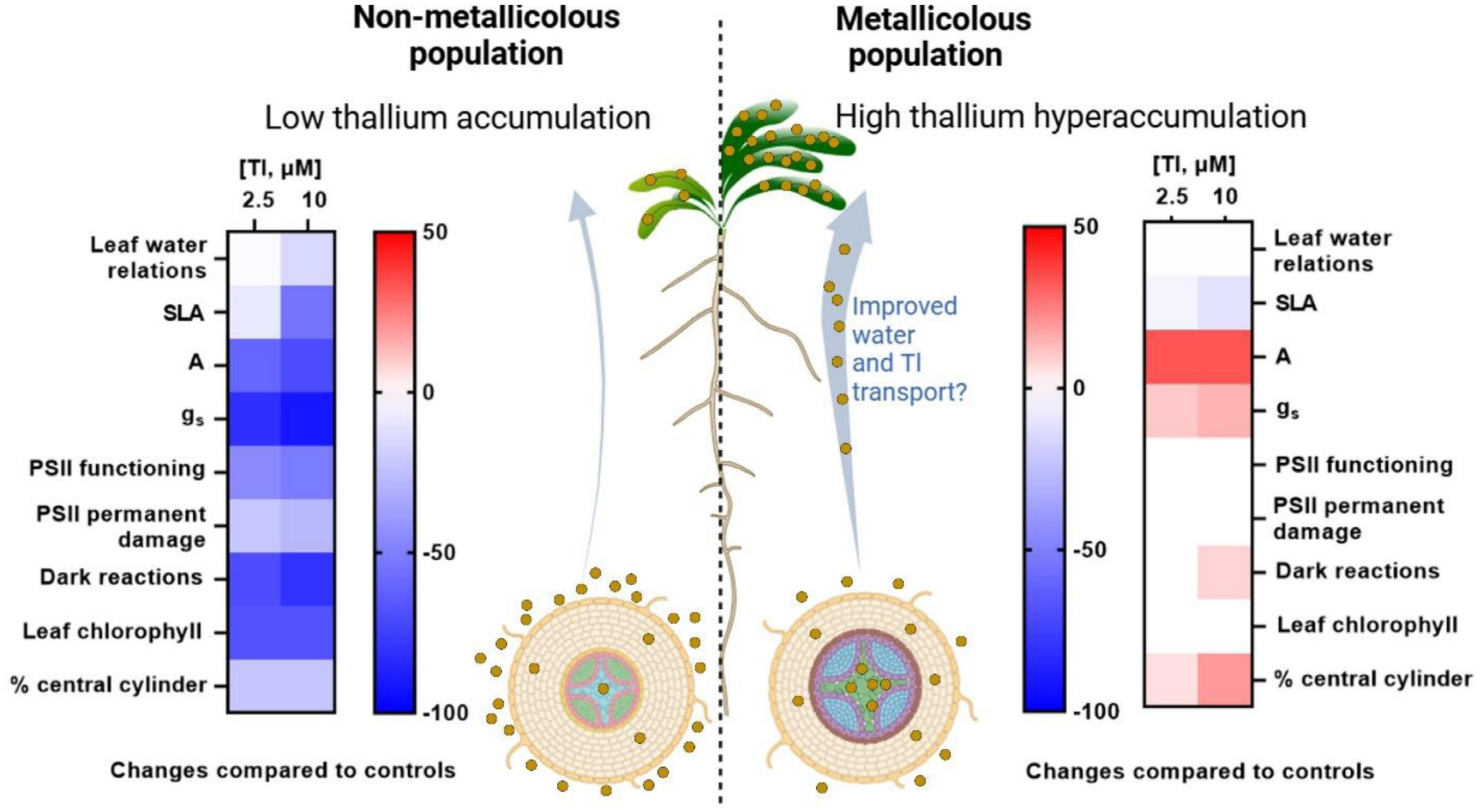
Schematic diagram illustrating the effects (% changes compared to controls) of Tl treatments on the two contrasting *S. latifolia* populations. While in the non‐metallicolous plants, Tl exposure impairs growth at all the studied levels; in the metallicolous plants, metal treatment has the opposite effect, improving stomatal conductance and thereby raising net CO_2_ assimilation rate and, probably, Tl accumulation in the leaves. Gas exchange and anatomical data indicate an improved hydraulic conductivity in the metallicolous population that would improve both water and thallium transport and translocation. The improved performance with Tl in the metallicolous plants suggests a beneficial role of this trace metal in this population. The diagram was made by BioRender.

Our study provides insights into Tl hyperaccumulating plants, with our data suggesting a positive effect of the hyperaccumulated element on plant photosynthetic performance and, consequently, its growth (Figure 13). Therefore, it opens avenues for the future identification of candidate genes associated with the mechanisms by which Tl enhances stomatal conductance and subsequently increases the net CO_2_ assimilation rate. How the plant can maintain stable photochemical reactions at impressively high foliar Tl concentrations remains another intriguing question.

## Author Contributions

**G.R.:** writing – review and editing, investigation, formal analysis. **I.B.:** writing – review and editing, investigation, formal analysis. **I.C.:** writing – review and editing, investigation, formal analysis. **E.C.:** writing – review and editing, investigation, formal analysis. **A.P.:** writing – review and editing, investigation, formal analysis. **M.D.:** writing – review and editing, investigation, formal analysis. **G.G.:** writing – review and editing, investigation, formal analysis. **A.v.d.E.:** writing – review and editing, conceptualization. **N.B.:** writing – review and editing, investigation, formal analysis, supervision. **C.G.:** writing – review and editing, writing – original draft, supervision, resources, conceptualization. All authors reviewed and approved the manuscript.

## Conflicts of Interest

The authors declare no conflicts of interest.

## Supporting information


**Figure S1:** Representative photographs of non‐metallicolous and metallicolous *S. latifolia* plants exposed to TlNO_3_ for 12 days. Non‐metallicolous population: (A) Control, (B) 2.5 μM Tl, (C) 10 μM Tl. Metallicolous population: (D) Control, (E) 2.5 μM Tl, (F) 10 μM Tl.
**Figure S2:** (A) palisade mesophyll thickness, (B) spongy mesophyll thickness, (C) percentage of palisade mesophyll thickness and (D) percentage of spongy mesophyll thickness of the non‐metallicolous and the metallicolous populations of *S. latifolia* exposed to TlNO_3_ for 12 days. Values are means ± SE of 12 replicates. Letters indicate the significant differences among samples according to the Tukey's test (at least *p* < 0.05), capital for inter‐population comparison and lower for intra‐population comparison.
**Figure S3:** Mesophyll cells of the non‐metallicolous population (A) and of the metallicolous population (B and C) in control conditions. (A) Chloroplasts with starch grains, grana stacks and a well‐shaped mitochondrion. (B) Mitochondrion near the vacuole with evident cristae. Numerous ribosomes can be observed close to the mitochondrion. (C) Chloroplast with well‐formed thylakoids and grana. V: vacuoles; mt: mitochondria; g: grana; tk: thylakoids; cw: cell wall. Scale bar: 500 nm (A); 200 nm (B, C).
**Figure S4:** Mesophyll cells of the non‐metallicolous population exposed to 10 μM Tl. (A) Portions of cytoplasm within the vacuole together with electron‐dense precipitates. (B) Electron‐dense zone in the cytoplasm resembling vacuole precipitates and part of a chloroplast with plastoglobuli. V: vacuoles; g: grana; tk: thylakoids; cw: cell wall; Ld: lipid droplet; VPs: precipitates; arrow: cytoplasm within the vacuole; arrowhead: plastoglobuli; asterisk: cytoplasm electron dense zone. Scale bar: 200 nm.
**Table S1:** Two‐way ANOVA results for increment in: (A, B) root length and (C, D) leaf area of the non‐metallicolous and the metallicolous populations of *S. latifolia* exposed to TlNO_3_ for 12 days.
**Table S2:** Two‐way ANOVA results for: (A) specific leaf area, (B) leaf water content and (C) leaf relative water content of the non‐metallicolous and the metallicolous populations of *S. latifolia* exposed to TlNO_3_ for 12 days.
**Table S3:** Two‐way ANOVA results for Tl concentration in: (A) roots and (B) shoots of the non‐metallicolous and the metallicolous populations of *S. latifolia* exposed to TlNO_3_ for 12 days.
**Table S4:** Two‐way ANOVA results for: (A, B) A_n_, (C, D) g_s_ and (E, F) C_i_ of the non‐metallicolous and the metallicolous populations of *S. latifolia* exposed to TlNO_3_ for 12 days.
**Table S5:** Two‐way ANOVA results for: (A, B) F_V_/F_M op_, (C, D), φPSII _op_, (E, F) ETR_op_, (G, H) NPQ_op_ and (I, J) Chlorophyll content index of the non‐metallicolous and the metallicolous populations of *S. latifolia* exposed to TlNO_3_ for 12 days.
**Table S6:** Two‐way ANOVA results for: (A) stomatal density upper surface, (B) stomatal density lower surface, (C) epidermal cell density upper surface, (D) epidermal cell density lower surface, (E) stomatal index upper surface, (F) stomatal index lower surface, (G) stomatal length upper surface, (H) stomatal length lower surface, (I) stomatal width upper surface and (J) stomatal width lower surface of the non‐metallicolous and the metallicolous populations of *S. latifolia* exposed to TlNO_3_ for 12 days.
**Table S7:** Two‐way ANOVA results for: (A) V_cmax_, (B) J_max_ and (C) TPU of the non‐metallicolous and the metallicolous populations of *S. latifolia* exposed to TlNO_3_ for 12 days.
**Table S8:** Two‐way ANOVA results for: (A) Photosynthetic efficiency and (B) Stomatal limitation of the non‐metallicolous and the metallicolous populations of *S. latifolia* exposed to TlNO_3_ for 12 days.
**Table S9:** Biometric, accumulation and photosynthetic parameters of the *S. latifolia* metallicolous populations exposed to low and high TlNO_3_ concentrations for 4 months. Values are mean of 6 replicates ± standard deviation. Letters indicate the significant differences among samples according to Tukey's test (at least *p* < 0.05), *F* and *p* values from one‐way ANOVA are reported.
**Table S10:** Two‐way ANOVA results for: (A) percentage of central cylinder area, (B) leaf thickness, (C) palisade mesophyll thickness, (D) spongy mesophyll thickness, (E) percentage of palisade mesophyll, (F) percentage of spongy mesophyll and (G) percentage of leaf intercellular spaces of the non‐metallicolous and the metallicolous populations of *S. latifolia* exposed to TlNO_3_ for 12 days.

## Data Availability

Data will be made available on request.
