# Supplementary material for: Physiological Effect of Thallium in the Facultative Hyperaccumulator Silene latifolia
Source: Physiol Plant. 2025 Aug 27;177(5):e70469. doi: 10.1111/ppl.70469 (PMC12382316; doi:10.1111/ppl.70469)

Supplementary material to

Physiological effect of thallium in the facultative hyperaccumulator *Silene latifolia*

Gaia Regini[1], Isabella Bettarini[1], Ilaria Colzi[1], Emilio Corti[1], Alessio Papini[1], Marco Dainelli[1], Giorgia Guardigli[1], Antony van der Ent[2], Nadia Bazihizina[1], Cristina Gonnelli[1*]

1) Department of Biology, Università degli Studi di Firenze, Via Micheli 1, 50121, Florence, Italy

2) Laboratory of Genetics, Wageningen University and Research, Droevendaalsesteeg 1, 6708 PB, Wageningen, The Netherlands

Fig S1: Representative photographs of non-metallicolous and metallicolous *S. latifolia* plants exposed to $TlNO_3$ for 12 days. Non-metallicolous population: A) Control, B) 2.5 µM Tl, C) 10 µM Tl. Metallicolous population: D) Control, E) 2.5 µM Tl, F) 10 µM Tl.

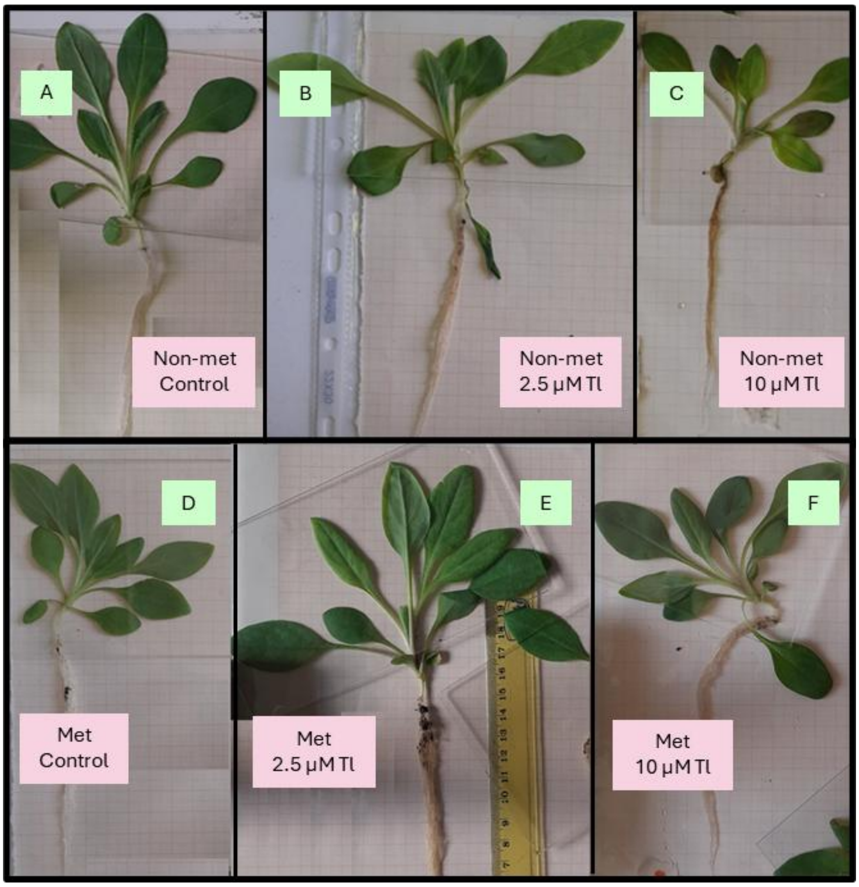

Table S1. Two-way ANOVA results for increment in: A-B) root length and C-D) leaf area of the non-metallicolous and the metallicolous populations of *S. latifolia* exposed to $TlNO_3$ for 12 days.

A) NON-MET	SS	DF	MS	F (DFn, DFd)	P value
Interaction	47.17	6	7.862	F (6, 132) = 3.354	P=0.0041
Time	536.7	3	178.9	F (3, 132) = 76.31	P<0.0001
Treatment	69.72	2	34.86	F (2, 132) = 14.87	P<0.0001
Residual	309.4	132	2.344		

B) MET	SS	DF	MS	F (DFn, DFd)	P value
Interaction	13.85	6	2.309	F (6, 132) = 11.446	P=0.2020
Time	8530.5	3	176.8	F (3, 132) = 110.7	P<0.0001
Treatment	1.216	2	0.6078	F (2, 132) = 0.3805	P=0.6843
Residual	210.8	132	1.597		

C) NON-MET	SS	DF	MS	F (DFn, DFd)	P value
Interaction	2288	6	381.4	F (6, 132) = 10.67	P<0.0001
Time	13260	3	4420	F (3, 132) = 123.6	P<0.0001
Treatment	3079	2	1539	F (2, 132) = 43.06	P<0.0001
Residual	4718	132	35.75		

D) MET	SS	DF	MS	F (DFn, DFd)	P value
Interaction	331.9	6	55.32	F (6, 132) = 1.879	P=0.0890
Time	18936	3	6312	F (3, 132) = 214.4	P<0.0001
Treatment	659.0	2	329.5	F (2, 132) = 11.19	P<0.0001
Residual	3886	132	29.44		

Table S2. Two-way ANOVA results for: A) specific leaf area, B) leaf water content and C) leaf relative water content of the non-metallicolous and the metallicolous populations of *S. latifolia* exposed to $TlNO_3$ for 12 days.

A) SLA	SS	DF	MS	F (DFn, DFd)	P value
Interaction	15209	2	7605	F (2, 66) = 3.543	P=0.0345
Treatment	60455	2	30227	F (2, 66) = 14.08	P<0.0001
Population	5209	1	5209	F (1, 66) = 2.427	P=0.1240
Residual	141658	66	2146		

B) WC	SS	DF	MS	F (DFn, DFd)	P value
Interaction	159.2	2	79.61	F (2, 66) = 6.689	P=0.0023
Treatment	97.57	2	48.79	F (2, 66) = 4.099	P=0.021
Population	219.6	1	219.6	F (1, 66) = 18.45	P<0.0001
Residual	758.5	66	11.90		

C) RWC	SS	DF	MS	F (DFn, DFd)	P value
Interaction	310.2	2	155.1	F (2, 66) = 3.175	P=0.0483
Treatment	748.7	2	347.4	F (2, 66) = 7.664	P=0.0010
Population	301.2	1	301.2	F (1, 66) = 6.165	P=0.0156
Residual	3224	66	48.85		

Table S3. Two-way ANOVA results for Tl concentration in: A) roots and B) shoots of the non-metallicolous and the metallicolous populations of *S. latifolia* exposed to $TlNO_3$ for 12 days.

A) roots	SS	DF	MS	F (DFn, DFd)	P value
Interaction	4165622	15	277708	F (15, 264) = 107.2	P<0.0001
Time	3206855	3	1068952	F (3, 264) = 412.7	P<0.0001
Treatment	8471558	5	1694312	F (5, 264) = 654.1	P<0.0001
Residual	683868	264	2590		

B) shoots	SS	DF	MS	F (DFn, DFd)	P value
Interaction	93581380	15	6238759	F (15, 264) = 4904	P<0.0001
Time	40092249	3	13364083	F (3, 264) = 10504	P<0.0001
Treatment	161782377	5	32356475	F (5, 264) = 25432	P<0.0001
Residual	335877	264	1272		

Table S4. Two-way ANOVA results for: A-B) A_n, C-D) g_s and E-F) C_i of the non-metallicolous and the metallicolous populations of *S. latifolia* exposed to $TlNO_3$ for 12 days.

A) NON-MET	SS	DF	MS	F (DFn, DFd)	P value
Interaction	53.41	6	8.902	F (6, 60) = 14.31	P<0.0001
Time	208.0	3	69.33	F (3, 60) = 111.5	P<0.0001
Treatment	57.29	2	28.65	F (2, 60) = 46.06	P<0.0001
Residual	37.32	60	0.6220		

B) MET	SS	DF	MS	F (DFn, DFd)	P value
Interaction	2.686	6	0.4477	F (6, 60) = 1.062	P=0.3955
Time	3.962	3	1.321	F (3, 60) = 3.133	P=0.0320
Treatment	4.277	2	2.139	F (2, 60) = 5.072	P=0.0092
Residual	25.30	60	0.4216		

C) NON-MET	SS	DF	MS	F (DFn, DFd)	P value
Interaction	316747	6	52791	F (6, 60) = 10.00	P<0.0001
Time	119729	3	39910	F (3, 60) = 7.563	P=0.0002
Treatment	578219	2	289110	F (2, 60) = 54.79	P<0.0001
Residual	316603	60	5277		

D) MET	SS	DF	MS	F (DFn, DFd)	P value
Interaction	76593	6	12765	F (6, 60) = 2.450	P=0.0348
Time	500726	3	166909	F (3, 60) = 32.03	P<0.0001
Treatment	123600	2	61800	F (2, 60) = 11.86	P<0.0001
Residual	312674	60	5211		

E) NON-MET	SS	DF	MS	F (DFn, DFd)	P value
Interaction	3131	6	521.8	F (6, 60) = 1.029	P=0.4158
Time	22177	3	7392	F (3, 60) = 14.57	P<0.0001
Treatment	3451	2	1725	F (2, 60) = 3.401	P=0.0399
Residual	30439	60	507.3		

F) MET	SS	DF	MS	F (DFn, DFd)	P value
Interaction	1922	6	320.3	$F(6, 60) = 1.645$	$P=0.1506$
Time	7303	3	2434	$F(3, 60) = 12.50$	$P<0.0001$
Treatment	101.2	2	50.58	$F(2, 60) = 0.2598$	$P=0.7721$
Residual	11683	60	194.7		

Table S5. Two-way ANOVA results for: A-B) $F_V/F_{M\ op}$, C-D), $\varphi PSII_{\ op}$ E-F) ETR_{op}, G-H) NPQ_{op} and I-J) Chlorophyll content index of the non-metallicolous and the metallicolous populations of *S. latifolia* exposed to $TlNO_3$ for 12 days.

A) NON-MET	SS	DF	MS	F (DFn, DFd)	P value
Interaction	0.08312	6	0.1385	F (6, 60) = 2.640	P=0.0244
Time	0.168	3	0.05599	F (3, 60) = 10.67	P<0.0001
Treatment	0.1279	2	0.06394	F (2, 60) = 12.19	P<0.0001
Residual	0.3148	60	0.005247		

B) MET	SS	DF	MS	F (DFn, DFd)	P value
Interaction	0.0001896	6	0.000031	F (6, 60) = 0.2868	P=0.9410
Time	0.01465	3	0.004885	F (3, 60) = 44.35	P<0.0001
Treatment	0.00000917	2	0.000004585	F (2, 60) = 0.04163	P=0.9583
Residual	0.006608	60	10.0001101		

C) NON-MET	SS	DF	MS	F (DFn, DFd)	P value
Interaction	0.2620	6	0.04367	F (6, 60) = 10.59	P<0.0001
Time	0.3188	3	0.1063	F (3, 60) = 25.77	P<0.0001
Treatment	0.3837	2	0.1919	F (2, 60) = 46.54	P<0.0001
Residual	0.2474	60	0.004123		

D) MET	SS	DF	MS	F (DFn, DFd)	P value
Interaction	0.0003979	6	0.00006632	F (6, 60) = 10.62	P=0.9954
Time	0.1128	3	0.03761	F (3, 60) = 60.24	P<0.0001
Treatment	0.0003511	2	0.0001755	F (2, 60) = 0.2812	P=0.7559
Residual	0.03746	60	0.0006243		

E) NON-MET	SS	DF	MS	F (DFn, DFd)	P value
Interaction	1730	6	288	F (6, 60) = 10.40	P<0.0001
Time	2225	3	741.6	F (3, 60) = 26.75	P<0.0001
Treatment	2604	2	1302	F (2, 60) = 46.95	P<0.0001
Residual	1664	60	27.73		

F) MET	SS	DF	MS	F (DFn, DFd)	P value
Interaction	14.89	6	2.482	F (6, 60) = 0.5266	P=0.7859
Time	803.9	3	268.0	F (3, 60) = 58.85	P<0.0001
Treatment	11.19	2	5.594	F (2, 60) = 1.187	P=0.3123
Residual	282.9	60	4.714		

G) NON-MET	SS	DF	MS	F (DFn, DFd)	P value
Interaction	0.2796	6	0.04660	F (6, 60) = 2.642	P=0.0243
Time	0.7855	3	0.2618	F (3, 60) = 14.84	P<0.0001
Treatment	0.4045	2	0.2023	F (2, 60) = 11.47	P<0.0001
Residual	1.058	60	0.01764		

H) MET	SS	DF	MS	F (DFn, DFd)	P value
Interaction	0.002852	6	0.0004753	F (6, 60) = 0.2310	P=0.9649
Time	0.04172	3	0.01391	F (3, 60) = 6.760	P=0.0005
Treatment	0.003409	2	0.001705	F (2, 60) = 0.8285	P=0.4416
Residual	0.1234	60	0.002057		

I) NON-MET	SS	DF	MS	F (DFn, DFd)	P value
Interaction	0.3931	6	0.06551	F (6, 60) = 8.443	P<0.0001
Time	0.009312	3	0.003104	F (3, 60) = 0.4000	P=0.7535
Treatment	0.6363	2	0.3181	F (2, 60) = 41.00	P<0.0001
Residual	0.4656	60	0.007760		

J) MET	SS	DF	MS	F (DFn, DFd)	P value
Interaction	0.02728	6	0.004547	F (6, 60) = 0.4452	P=0.8455
Time	0..2285	3	0.07615	F (3, 60) = 7.457	P=0.0003
Treatment	0.03289	2	0.01645	F (2, 60) = 1.610	P=0.2083
Residual	0.6127	60	0.01021		

Table S6. Two-way ANOVA results for: A) stomatal density upper surface, B) stomatal density lower surface, C) epidermal cell density upper surface, D) epidermal cell density lower surface, E) stomatal index upper surface, F) stomatal index lower surface, G) stomatal length upper surface, H) stomatal length lower surface, I) stomatal width upper surface and J) stomatal width lower surface of the non-metallicolous and the metallicolous populations of *S. latifolia* exposed to $TlNO_3$ for 12 days.

A)	SS	DF	MS	F (DFn, DFd)	P value
Interaction	383.2	2	191.6	F (2, 66) = 3.806	P=0.0273
Treatment	139.8	2	69.89	F (2, 66) = 1.388	P=0.2566
Population	130.4	1	130.4	F (1, 66) = 2.590	P=0.1123
Residual	3322	66	50.34		

B)	SS	DF	MS	F (DFn, DFd)	P value
Interaction	7219	2	3609	F (2, 66) = 16.46	P<0.0001
Treatment	1122	2	560.9	F (2, 66) = 2.558	P=0.0852
Population	2393	1	2393	F (1, 66) = 10.91	P=0.0015
Residual	14475	66	219.3		

C)	SS	DF	MS	F (DFn, DFd)	P value
Interaction	13051	2	6526	F (2, 66) = 5.640	P=0.0055
Treatment	6167	2	3083	F (2, 66) = 2.665	P=0.0771
Population	24421	1	24421	F (1, 66) = 21.11	P<0.0001
Residual	76359	66	1157		

D)	SS	DF	MS	F (DFn, DFd)	P value
Interaction	42562	2	21281	F (2, 66) = 11.37	P<0.0001
Treatment	13401	2	6701	F (2, 66) = 3.580	P=0.0334
Population	5956	1	5956	F (1, 66) = 3.182	P=0.0791
Residual	123549	66	1872		

E)	SS	DF	MS	F (DFn, DFd)	P value
Interaction	36.19	2	18.10	F (2, 66) = 1.231	P=0.02986
Treatment	2.307	2	1.153	F (2, 66) = 0.07845	P=0.9246
Population	68.11	1	68.11	F (1, 66) = 4.633	P=0.0350
Residual	970.3	66	14.70		

F)	SS	DF	MS	F (DFn, DFd)	P value
Interaction	72.92	2	36.46	F (2, 66) = 4.683	P=0.0125
Treatment	17.83	2	8.914	F (2, 66) = 1.145	P=0.3245
Population	13.05	1	13.05	F (1, 66) = 1.676	P=0.1999
Residual	513.9	66	7.786		

G)	SS	DF	MS	F (DFn, DFd)	P value
Interaction	163.6	2	81.82	F (2, 66) = 4.375	P=0.0164
Treatment	52.16	2	26.08	F (2, 66) = 1.394	P=0.2552
Population	77.43	1	77.43	F (1, 66) = 4.140	P=0.0459
Residual	1234	66	18.43		

H)	SS	DF	MS	F (DFn, DFd)	P value
Interaction	260.4	2	130.2	F (2, 66) = 6.306	P=0.0031
Treatment	16.26	2	8.130	F (2, 66) = 0.3937	P=0.6761
Population	14.43	1	14.43	F (1, 66) = 0.6989	P=0.4062
Residual	1363	66	20.65		

I)	SS	DF	MS	F (DFn, DFd)	P value
Interaction	21.01	2	10.51	F (2, 66) = 4.188	P=0.0194
Treatment	11.56	2	5.779	F (2, 66) = 2.302	P=0.1080
Population	2.493	1	2.493	F (1, 66) = 0.9933	P=0.3226
Residual	165.7	66	2.510		

J)	SS	DF	MS	F (DFn, DFd)	P value
Interaction	38.53	2	19.26	F (2, 66) = 2.164	P=0.1230
Treatment	23.70	2	11.85	F (2, 66) = 1.331	P=0.2712
Population	0.01386	1	0.01386	F (1, 66) = 0.001557	P=0.9686
Residual	587.6	66	8.903		

Table S7. Two-way ANOVA results for: A) V_{cmax}, B) J_{max} and C) TPU of the non-metallicolous and the metallicolous populations of *S. latifolia* exposed to $TlNO_3$ for 12 days.

A)	SS	DF	MS	F (DFn, DFd)	P value
Interaction	9011	2	4505	F (2, 30) = 120.8	P<0.0001
Treatment	5325	2	2662	F (2, 30) = 71.37	P<0.0001
Population	10272	1	10272	F (1, 30) = 275.4	P<0.0001
Residual	1119	30	37.30		

B)	SS	DF	MS	F (DFn, DFd)	P value
Interaction	19761	2	9880	F (2, 30) = 76.37	P<0.0001
Treatment	12778	2	6389	F (2, 30) = 49.38	P<0.0001
Population	21067	1	21067	F (1, 30) = 162.8	P<0.0001
Residual	3881	30	129.4		

C)	SS	DF	MS	F (DFn, DFd)	P value
Interaction	55.1	2	27.55	F (2, 30) = 53.12	P<0.0001
Treatment	31.79	2	15.89	F (2, 30) = 30.65	P<0.0001
Population	61.89	1	61.89	F (1, 30) = 119.3	P<0.0001
Residual	15.56	30	0.5186		

Table S8. Two-way ANOVA results for: A) Photosynthetic efficiency and B) Stomatal limitation of the non-metallicolous and the metallicolous populations of *S. latifolia* exposed to $TlNO_3$ for 12 days.

A)	SS	DF	MS	F (DFn, DFd)	P value
Interaction	0.003488	2	0.001744	F (2, 30) = 29.73	P<0.0001
Treatment	0.002194	2	0.001097	F (2, 30) = 18.70	P<0.0001
Population	0.004472	1	0.004472	F (1, 30) = 76.22	P<0.0001
Residual	0.001760	30	0.00005867		

B)	SS	DF	MS	F (DFn, DFd)	P value
Interaction	244.1	2	122.0	F (2, 30) = 11.02	P=0.0003
Treatment	622.1	2	311.0	F (2, 30) = 28.09	P<0.0001
Population	14.36	1	14.36	F (1, 30) = 1.297	P=0.2637
Residual	332.2	30	11.07		

Table S9. Biometric, accumulation and photosynthetic parameters of the *S. latifolia* metallicolous populations exposed to low and high $TlNO_3$ concentrations for 4 months. Values are mean of 6 replicates ± standard deviation. Letters indicate the significant differences among samples according to Tukey's test (at least p<0.05), F and p values from one-way ANOVA are reported.

	**0**	**Low Tl**	**High Tl**	**F**	**p**
**Root fresh weight (g)**	31.1 ± 4.2 **b**	28.4 ± 2.1 **b**	25.8 ± 3.9 **a**	3.393	0.0493
**Shoot fresh weight (g)**	43.2 ± 3.7 **b**	41.8 ± 4.2 **b**	37.5 ± 3.3 **a**	3.762	0.0474
**Root dry weigh (g)**	2.8 ± 0.2 **b**	2.7 ± 0.8 **b**	2.0 ± 0.3 **a**	4.442	0.0305
**Shoot dry weight (g)**	10.6 ± 1.1 **b**	10.1 ± 0.7 **b**	9.2 ± 0.8 **a**	3.872	0.0441
**Root Tl (µg g^{-1} d.w.)**	bdl	2750 ± 470	3600 ± 730		
**Shoot Tl (µg g^{-1} d.w.)**	bdl	4060 ± 780	15670 ± 1340		
**SLA (cm^2 g^{-1})**	136 ± 12 **a**	114 ± 27 **a**	118 ± 15 **a**	2.248	0.1400
**Leaf WC (%)**	80.0 ± 2.3 **a**	80.3 ± 4.4 **a**	80.2 ± 3.7 **a**	0.0108	0.9892
**Leaf RWC (%)**	85.5 ± 2.3 **a**	86.9 ± 4.6 **a**	85.2 ± 1.3 **a**	0.5913	0.5660
**A_{op} (µmol m^{-2} s^{-1})**	4.4 ± 0.5 **b**	3.6 ± 0.7 **b**	3.5 ± 0.4 **a**	4.867	0.0235
**$G_{s\ op}$ (mmol m^{-2} s^{-1})**	207 ± 40 **b**	174 ± 39 **ab**	152 ± 29 **a**	3.482	0.0491
**$C_{i\ op}$ (µmol mol^{-1})**	342 ± 11 **a**	364 ± 14 **a**	341 ± 20 **a**	4.243	0.0547
**$F_V/F_{M\ op}$**	0.83 ±0.01 **a**	0.82 ± 0.01 **a**	0.82 ± 0.01 **a**	2.000	0.1698
**$\varphi PSII_{op}$**	0.69 ± 0.04 **a**	0.65 ± 0.01 **a**	0.63 ± 0.06 **a**	3.170	0.0711
**ETR_{op} (µmol e^- m^{-2} s^{-1})**	57.8 ± 2.7 **a**	54.4 ± 1.3 **a**	52.9 ± 4.9 **a**	3.439	0.0590
**NPQ_{op}**	0.27 ± 0.04 **a**	0.35 ± 0.06 **a**	0.36 ± 0.1 **a**	2.882	0.0873
**Chl index**	1.04 ± 0.1 **a**	1.1 ± 0.2 **a**	0.9 ± 0.1 **a**	3.160	0.0716
**V_{cmax} (µmol CO_2 m^{-2} s^{-1})**	62.6 ± 3.5 **b**	63.8 ± 6.1 **b**	42.5 ± 10.8 **a**	15.52	0.0002
**J_{max} (µmol e^- m^{-2} s^{-1})**	118 ± 12 **b**	112 ± 22 **ab**	88 ± 15 **a**	5.318	0.0180
**TPU (µmol Pi m^{-2} s^{-1})**	7.8 ± 0.6 **b**	7.5 ± 1.0 **ab**	6.7 ± 0.5 **a**	3.615	0.497

**Photosynthetic efficiency (A/Ci)**	0.043 ± 0.007 **b**	0.033 ± 0.008 **ab**	0.024 ± 0.011 **a**	6.949	0.073
**Stomatal limitation (%)**	41.3 ± 3.8 **a**	45.1 ± 2.7 **ab**	49.8 ± 8.0 **b**	3.807	0.046

Table S10. Two-way ANOVA results for: A) percentage of central cylinder area, B) leaf thickness, C) palisade mesophyll thickness, D) spongy mesophyll thickness, E) percentage of palisade mesophyll, F) percentage of spongy mesophyll and G) percentage of leaf intercellular spaces of the non-metallicolous and the metallicolous populations of *S. latifolia* exposed to $TlNO_3$ for 12 days.

A)	SS	DF	MS	F (DFn, DFd)	P value
Interaction	17.66	2	8.831	F (2, 66) = 25.46	P<0.0001
Treatment	1.952	2	0.9761	F (2, 66) = 2.814	P=0.0672
Population	0.3058	1	0.3058	F (1, 66) = 0.8815	P=0.3512
Residual	22.90	66	0.3469		

B)	SS	DF	MS	F (DFn, DFd)	P value
Interaction	15538	2	7769	F (2, 66) = 10.68	P<0.0001
Treatment	14465	2	7232	F (2, 66) = 9.942	P=0.0002
Population	392.4	1	392.4	F (1, 66) = 0.5394	P=0.4653
Residual	48014	66	727.5		

C)	SS	DF	MS	F (DFn, DFd)	P value
Interaction	1576	2	787.8	F (2, 66) = 8.283	P=0.0006
Treatment	2299	2	1149	F (2, 66) = 12.08	P<0.0001
Population	1406	1	1406	F (1, 66) = 14.78	P=0.0003
Residual	6278	66	95.12		

D)	SS	DF	MS	F (DFn, DFd)	P value
Interaction	786.9	2	393.4	F (2, 66) = 3.944	P=0.0241
Treatment	2586	2	1293	F (2, 66) = 12.96	P<0.0001
Population	8448	1	8448	F (1, 66) = 84.68	P<0.0001
Residual	6584	66	99.76		

E)	SS	DF	MS	F (DFn, DFd)	P value
Interaction	76.96	2	38.48	F (2, 66) = 5.266	P=0.0076
Treatment	73.54	2	36.77	F (2, 66) = 5.032	P=0.0092
Population	1371	1	1371	F (1, 66) = 187.6	P<0.0001
Residual	482.3	66	7.307		

F)	SS	DF	MS	F (DFn, DFd)	P value
Interaction	65.37	2	32.68	F (2, 66) = 4.395	P=0.0161
Treatment	62.19	2	31.10	F (2, 66) = 4.182	P=0.0195
Population	1371	1	1371	F (1, 66) = 184.4	P<0.0001
Residual	490.8	66	7.436		

G)	SS	DF	MS	F (DFn, DFd)	P value
Interaction	17.66	2	8.831	F (2, 66) = 25.46	P<0.0001
Treatment	1.952	2	0.9761	F (2, 66) = 2.814	P=0.0672
Population	0.3058	1	0.3058	F (1, 66) = 0.8815	P=0.3512
Residual	22.90	66	0.3469		

Fig S2: A) palisade mesophyll thickness, B) spongy mesophyll thickness, C) percentage of palisade mesophyll thickness and D) percentage of spongy mesophyll thickness of the non-metallicolous and the metallicolous populations of *S. latifolia* exposed to $TlNO_3$ for 12 days. Values are means ± SE of 12 replicates. Letters indicate the significant differences among samples according to the Tukey's test (at least $p < 0.05$), capital for inter-population comparison and lower for intra-population comparison.

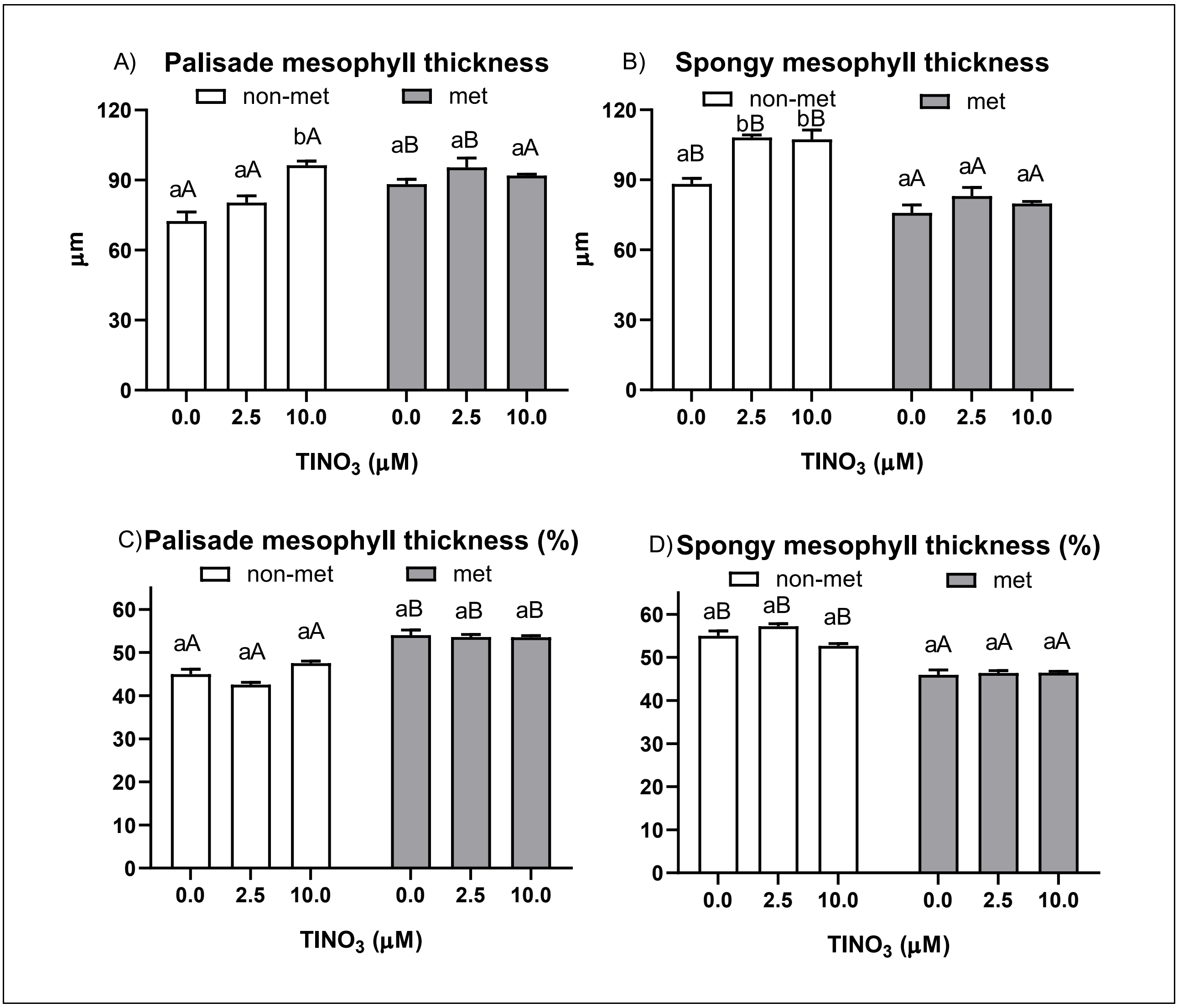

Fig. S3. Mesophyll cells of the non-metallicolous population (A) and of the metallicolous population (B and C) in control conditions. A) Chloroplasts with starch grains, grana stacks and a well-shaped mitochondrion. B) Mitochondrion near the vacuole with evident cristae. Numerous ribosomes can be observed close to the mitochondrion. C) Chloroplast with well-formed thylakoids and grana. V: vacuoles; mt: mitochondria; g: grana; tk: thylakoids; cw: cell wall. Scale bar: 500 nm (A); 200 nm (B, C).

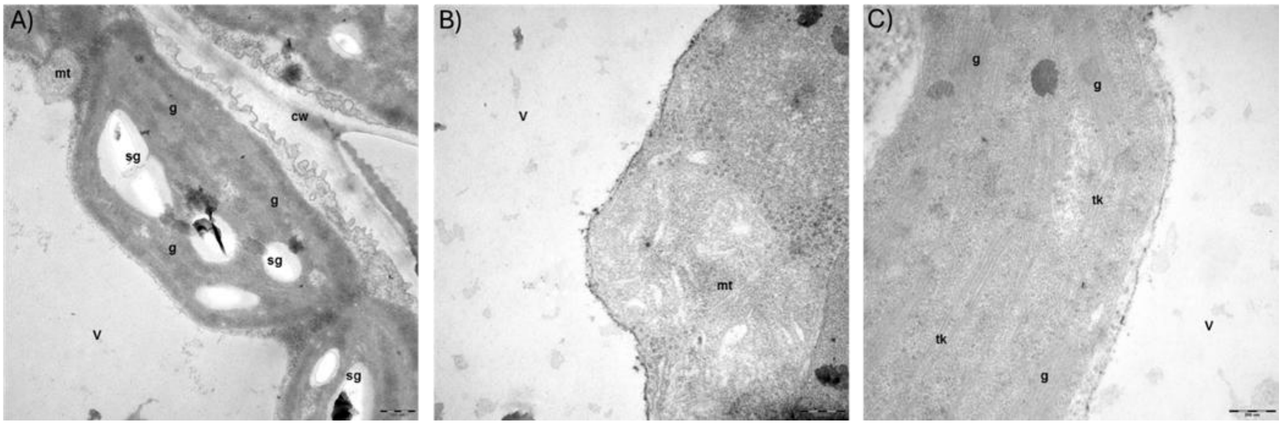

Fig. S4. Mesophyll cells of the non-metallicolous population exposed to 10μM Tl. A) Portions of cytoplasm within the vacuole together with electron-dense precipitates. B) Electron-dense zone in the cytoplasm resembling vacuole precipitates, and part of a chloroplast with plastoglobuli. V: vacuoles; g: grana; tk: thylakoids; cw: cell wall; Ld: lipid droplet; VPs: precipitates; arrow: cytoplasm within the vacuole; arrowhead: plastoglobuli; asterisk: cytoplasm electron dense zone. Scale bar: 200 nm.

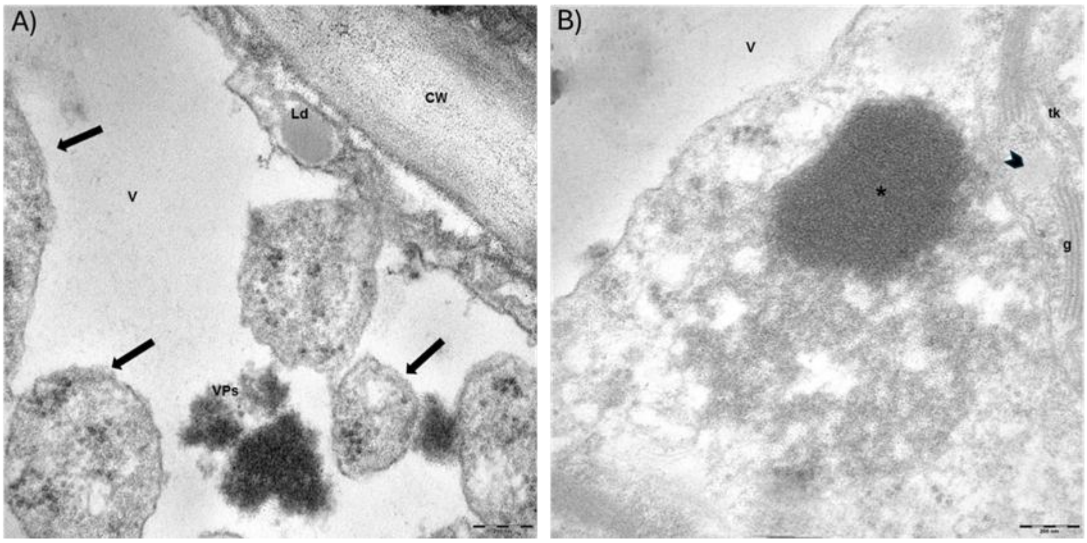

Supplement: Supplementary file 1 — Figure S1: Representative photographs of non‐metallicolous and metallicolous S. latifolia plants exposed to TlNO3 for 12 days. Non‐metallicolous population: (A) Control, (B) 2.5 μM Tl, (C) 10 μM Tl. Metallicolous population: (D) Control, (E) 2.5 μM Tl, (F) 10 μM Tl. Figure S2: (A) palisade mesophyll thickness, (B) spongy mesophyll thickness, (C) percentage of palisade mesophyll thickness and (D) percentage of spongy mesophyll thickness of the non‐metallicolous and the metallicolous populations of S. latifolia exposed to TlNO3 for 12 days. Values are means ± SE of 12 replicates. Letters indicate the significant differences among samples according to the Tukey's test (at least p < 0.05), capital for inter‐population comparison and lower for intra‐population comparison. Figure S3: Mesophyll cells of the non‐metallicolous population (A) and of the metallicolous population (B and C) in control conditions. (A) Chloroplasts with starch grains, grana stacks and a well‐shaped mitochondrion. (B) Mitochondrion near the vacuole with evident cristae. Numerous ribosomes can be observed close to the mitochondrion. (C) Chloroplast with well‐formed thylakoids and grana. V: vacuoles; mt: mitochondria; g: grana; tk: thylakoids; cw: cell wall. Scale bar: 500 nm (A); 200 nm (B, C). Figure S4: Mesophyll cells of the non‐metallicolous population exposed to 10 μM Tl. (A) Portions of cytoplasm within the vacuole together with electron‐dense precipitates. (B) Electron‐dense zone in the cytoplasm resembling vacuole precipitates and part of a chloroplast with plastoglobuli. V: vacuoles; g: grana; tk: thylakoids; cw: cell wall; Ld: lipid droplet; VPs: precipitates; arrow: cytoplasm within the vacuole; arrowhead: plastoglobuli; asterisk: cytoplasm electron dense zone. Scale bar: 200 nm. Table S1: Two‐way ANOVA results for increment in: (A, B) root length and (C, D) leaf area of the non‐metallicolous and the metallicolous populations of S. latifolia exposed to TlNO3 for 12 days. Table S2: Two‐way [file PPL-177-e70469-s001.pdf]
